# Advancements and Challenges in Tissue-Engineered Heart Valves: Integrating Biomechanics, Biomaterials, and Biomimetic Design for Functional Maturity

**DOI:** 10.3390/biomimetics11030185

**Published:** 2026-03-04

**Authors:** Lorenzo Guidi, Elisabetta Rosellini, Gaia Riccio, Maria Grazia Cascone

**Affiliations:** Department of Civil and Industrial Engineering, University of Pisa, 56126 Pisa, Italy; lorenzo.guidi@phd.unipi.it (L.G.); elisabetta.rosellini@unipi.it (E.R.); gaia.riccio@ing.unipi.it (G.R.)

**Keywords:** tissue-engineered heart valves, biomimetic design, heart valve mechanobiology, scaffold architecture, biological cues, bioreactors, functional maturation

## Abstract

Valvular heart disease remains a major global health burden, with currently available prosthetic heart valves failing to fully reproduce the adaptive, regenerative, and long-term functional properties of native valves. Tissue-engineered heart valves (TEHVs) have emerged as a promising alternative, aiming to develop living valve replacements capable of growth, remodeling, and physiological integration. However, despite substantial progress, the clinical translation of TEHVs remains limited, indicating the need for design strategies that go beyond material selection toward functionally mature constructs. This review presents recent advances in TEHV development from a biomimetic perspective, using native heart valves as a biological reference characterized by hierarchical structure, anisotropic mechanical behavior, mechanoresponsive cell populations, immune regulation, and temporally coordinated remodeling. We integrate current understanding of valve biology and mechanobiology with advances in scaffold materials and architecture, bioactive functionalization, biomechanical conditioning, and emerging fabrication and monitoring technologies. We discuss how biomimetic scaffold designs aim to replicate native extracellular matrix organization and nonlinear mechanics, how biological cues are used to regulate thrombosis, immune response, and cell recruitment, and how dynamic bioreactor systems support functional tissue maturation through controlled mechanical stimulation. Finally, key challenges for clinical translation are highlighted, and future directions are outlined, emphasizing integrated and biomimetically informed design approaches. Overall, this review aims to define guiding principles that may accelerate the development of durable, regenerative, and clinically translatable tissue-engineered heart valves. We argue that successful TEHV translation requires synchronized control of scaffold anisotropy, immune modulation, and mechanical conditioning rather than incremental material optimization.

## 1. Introduction

Valvular heart disease (VHD) represents a growing global health challenge, driven particularly by aging populations, lifestyle-conditions and pathologies such as diabetes or hypertension [1,2,3]. According to population-based studies, the overall prevalence of moderate to severe VHD in industrialized countries is about 2.5% of the general adult population, rising sharply with age [1,4]. The clinical burden is multifaceted: VHD contributes not only to high mortality but also to heart failure, arrhythmias, reductions in quality of life and significant healthcare costs [1,2,3,4,5].

To treat severe VHD, especially when the disease is symptomatic or causes significant functional impairment, valve replacement is the standard of care [3,6]. Two principal types of prosthetic heart valves are commonly used: mechanical and bioprosthetic valves. Mechanical valves are currently available in a variety of shapes, sizes and materials, and are the gold standard treatment for patients up to approximately 70 years old. They have the advantage of durability with an average life span of over 20 years [6]. However, regardless of the type of mechanical valve implanted, they necessitate lifelong anticoagulation therapy and thus impose an increased risk of bleeding or strokes, as well as lifestyle limitations for patients [3,4,5,6,7]. To reduce the thromboembolic complications of the mechanical valves, bioprosthetic valves, based on xenograft, allograft or homograft, have been introduced. Bioprosthetic valves’ structure and geometry resemble the native valve more closely than the mechanical ones, resulting in better hemodynamic and reduced thrombus formation, avoiding the need for permanent anticoagulation therapy. However, the use of xenogenic or allogenic materials increases the risk of immunogenic reactions and disease transmission. In addition, their durability is substantially lower: structural valve deterioration (SVD), calcification, leaflet tearing or degeneration often leads to valve dysfunction and need for reoperation, especially in young patients [3,4,5,6,7].

Conventional prosthetic valves, whether mechanical or bioprosthetic, fail to reproduce the complex functional, mechanical, biological, and adaptive characteristics of native heart valves. In addition, these types of valve replacement lack growth, repair, and remodeling potential. An ideal heart valve substitute should therefore not only restore unidirectional blood flow, but also adapt to dynamic hemodynamic loads, support endothelialization and extracellular matrix (ECM) turnover, minimize thrombogenicity, resist calcification and mechanical fatigue, and exhibit long-term biocompatibility and immunological tolerance. These requirements are particularly critical in pediatric patients, where the inability of current prostheses to accommodate somatic growth often necessitates repeated surgical interventions [2,8,9].

Considering these limitations, tissue engineered heart valves (TEHVs) have emerged as a promising alternative. THEVs are designed to replicate the architecture and function of natural heart valves through various tissue engineering (TE) approaches (in vitro, in vivo and in situ), utilizing living cells and biomaterials to generate constructs with regenerative proprieties. Heart valve tissue engineering employs a wide range of biomaterials and biofabrication strategies such as molding, electrospinning, melt electrowriting and 3D bioprinting to create either cellular or cell-free bioresorbable constructs that can ultimately yield living functional valves in vivo [3].

These approaches differ in complexity, level of biological control, and translational readiness. The in vitro TE paradigm, originally defined by Langer and Vacanti in 1993, is based on scaffold seeding with autologous cells, in vitro tissue maturation within a bioreactor and subsequent in vivo remodeling following implantation [3,10]. Key processes include cell proliferation and migration, ECM production and organization and scaffold degradation, all of which must be tightly balanced to achieve appropriate tissue maturation over time [6].

The in vivo TE approach instead exploits the human body as a bioreactor relying on tissue encapsulation of a nondegradable scaffold following subcutaneous implantation to generate a collagen-rich matrix that can later be harvested and used as an autologous replacement. While this strategy enables the formation of non-immunogenic tissue, it is limited by poor control over tissue thickness, intrinsic thrombogenicity, lack of elastin, and the need for invasive procedures and long implantation times [6].

More recently, the in situ TE approach has gained increasing attention as a potentially simpler and more cost-effective strategy for producing off-the-shelf implants. This approach involves the direct implantation of a cell-free scaffold at the native valve site, leveraging the body’s intrinsic regenerative capacity to recruit endogenous cells and promote tissue formation and remodeling while providing initial mechanical functionality [3,6].

Despite significant progress across these paradigms, the clinical translation of TEHVs remains limited, indicating that technological advancement alone is insufficient. Increasing evidence suggests that successful valve regeneration critically depends on how effectively engineered constructs recapitulate the multiscale principles governing native valve function, including hierarchical structure, anisotropic mechanics, mechanoresponsive cell behavior, immune regulation, and temporally coordinated remodeling.

In this review, we critically examine recent advances in tissue-engineered heart valves through the lens of biomimetics. By integrating insights from native valve biology and mechanobiology with contemporary strategies in scaffold design, bioactive functionalization, biomechanical conditioning, and emerging technologies, we aim to clarify how biomimetic principles can guide the development of functionally mature TEHVs. Furthermore, we discuss current challenges toward clinical translation and highlight future directions for achieving durable, regenerative, and clinically viable heart valve replacements. While numerous reviews address individual aspects of TEHV development (materials, cells, or bioreactors), fewer integrate mechanobiology, immune regulation, and scaffold architecture as a coupled system. This review specifically addresses how failure to synchronize these dimensions underlies limited clinical translation.

## 2. Native Heart Valve Structure and Pathophysiology

### 2.1. The Anatomy of Healthy Heart Valves

Human heart valves are highly specialized, dynamic structures whose primary function is to ensure unidirectional blood flow through more than 100,000 opening and closing cycles per day, and approximately 3 billion cycles over a lifetime [11,12,13]. The human heart contains four valves—two atrioventricular valves (AV), the mitral and tricuspid valves—that separate the atria from the ventricles, and two semilunar valves (SL), the aortic and pulmonary valves, that separate the ventricles from the great arteries [4,12]. Each valve is tailored to the hemodynamic conditions of its anatomical location. The AV consist of two (mitral) or three (tricuspid) leaflets, with external supporting chordae tendineae that attach the leaflet to papillary muscles within the ventricles. In contrast, the SVs comprise three leaflets termed cusps and lack an external chordae and papillary muscles [4,11].

In diastole, the papillary muscles are relaxed, and high pressure in the atrium causes opening of the mitral (left) and tricuspid (right) valve leaflets to promote blood flow into the respective ventricle. Once ventricular pressure increases, the AV leaflets close and maintain coaptation to prevent eversion of the valve leaflets into the atria. As the ventricle contracts, blood exits through the open SL and the ventricle relaxes to begin the cycle again [11].

The architecture of the valves reflects their functional demands. The leaflets and cusps are composed of a stratified and anisotropic extracellular matrix (ECM) organized into three distinct layers [4,11,12]:

- The fibrosa (~45%), located on the outflow side, is composed of densely collagen fibers (mainly type 1), aligned circumferentially. This layer confers tensile strength and resistance to high transvalvular pressures, especially in the aortic and mitral valves, during valve closure. Due to the high degree of collagen alignment, the fibrosa represents the primary load-bearing layer of the leaflet.
- The spongiosa (~35%), forms the middle layer, which accommodates relative motions between adjacent layers. It is composed mainly of proteoglycans (primarily Versican) and glycosaminoglycans (GAG) including hyaluronan, which provide viscoelastic damping, resist compression and contribute to leaflet flexibility.
- The ventricularis (or atrialis) (~20%), on the inflow side, is dominated by elastin fibers aligned radially, facilitating stretch and retraction, enabling rapid leaflet opening and restoration of leaflet geometry after each cardiac cycle.

This layered ECM structure ensures that stiffness and flexibility are finely balanced across different loading conditions during the open closure cycle [12]. Importantly, leaflet architecture varies between valves to match their pressure and loading environment: the left-sided valves (aortic and mitral) endure significantly higher pressures than the right-sided valves. As a result, the aortic and mitral valves are structurally more robust than their right-sided counterparts [12].

The ECM components of the valve leaflet are populated by valvular interstitial cells (VICs) and encapsulated by an overlying single layer of valve endothelial cells (VECs). VICs are distributed throughout the ECM and are primarily responsible for ECM synthesis, turnover and repair, acting as dynamic regulators of matrix composition and mechanical integrity. This is achieved by a balanced secretion of matrix degradation enzymes, including matrix metalloproteinases (MMPs), and their inhibitors (tissue inhibitor of metalloproteinase TIMPs). On the other hand, VECs form a tight and functional barrier between the blood and the inner valve tissue, protecting it against the physical and mechanical stress of the hemodynamic environment, preventing inflammation and thrombosis. In addition, VECs have been shown to communicate molecularly with VICs by sensing hemodynamic forces and translating them into biochemical signals that regulate VIC behavior [5,11,12].

### 2.2. Mechanobiology of Native Valves

Native heart valves are exposed to a complex system of mechanical forces, which vary spatially and temporally throughout the cardiac cycle. These include [11,12]:

- Shear stress, generated by blood flow over the leaflet surface, which is laminar and unidirectional on the ventricularis/atrialis layer but oscillatory and disturbed on the fibrosa layer [14,15].
- Tensile stress, experienced during valve closure, especially affects the fibrosa layer.
- Cyclic flexural loading, inherent to the repetitive opening and closing cycles, that challenges leaflet durability and requires elastin-mediated resilience.

Both VICs and VECs are highly mechanoresponsive. VECs respond to shear stress by regulating nitric oxide production, adhesion molecule expression, and secretion of paracrine mediators that influence VIC phenotype [5,16,17]. VICs, in turn, remodel the ECM in response to cyclic strain, balancing matrix synthesis and degradation via MMPs and TIMPs. In health valves, VICs are in a quiescent state. However, during growth, remodeling, or injury, VICs can transiently differentiate into an activated myofibroblast-like phenotype, characterized by proliferation, apoptosis and expression of α-smooth muscle actin. This activated state enables ECM deposition and tissue remodeling [5]. Disruption of this finely tuned mechanobiological balance, promotes maladaptive remodeling. Thus, mechanobiology represents not only a cornerstone of normal valve homeostasis, but also a key driver of pathological processes when physiological mechanical forces are varied.

### 2.3. Pathological Remodeling

Valvular heart disease arises when the tightly regulated structure–function–mechanobiology balance of the valve is disrupted, leading to maladaptive remodeling of leaflet tissue [11,12]. Abnormal mechanical loading, genetic predisposition, and inflammatory stimuli can trigger pathological changes in extracellular matrix organization and cellular phenotype. These processes are commonly associated with three major remodeling outcomes: myxomatous degeneration, characterized by proteoglycan accumulation and collagen disruption; fibrotic remodeling, involving excessive collagen deposition and leaflet stiffening; and calcification, in which VICs undergo osteogenic differentiation and deposit mineralized nodules within the ECM [11,12].

At the cellular level, pathological remodeling is largely driven by sustained VIC activation and dysregulated matrix turnover, ultimately compromising leaflet mechanics, durability, and valve function.

## 3. Biomimetic Strategies in Scaffold Design

Building on the structural organization, mechanobiological behavior, and failure mechanisms of native heart valves, biomimetic scaffold design aims to translate these features into well-defined and controllable engineering strategies. Heart valves are characterized by a highly complex and articulated structure, upon which valve leaflet function critically depends. The primary design objective for TEHVs is therefore to mimic the native tissue microstructure, while matching native mechanical proprieties and anisotropy [4], ensuring adequate strength, flexibility and durability to endure the cyclic stresses and strains of the cardiovascular environment. Accordingly, scaffold design and properties play a crucial role in the success of both in vitro and in situ TEHVs [6]. One of the key considerations in the design of polymeric scaffolds for TEHVs is the biodegradation profile. Ideally, degradation byproducts should be biocompatible and non-toxic, and the rate of degradation should be coordinated with new tissue formation, thereby preserving structural integrity while providing support for progressive tissue formation, for in situ TEHVs [3,6,7].

If the scaffold degrades too rapidly, valve function may be compromised, and infiltrating cells may lose the guiding template for regeneration. On the other hand, excessively slow scaffold degradation may induce prolonged inflammatory responses and impair remodeling [5,6]. In addition, scaffolds should exhibit appropriate porosity and interconnectivity to favor cell infiltration, nutrient transport and neovascularization. They should also provide nano- and microscale cues capable of regulating cell adhesion, proliferation and phenotype, while ensuring hemocompatibility and resistance to calcification, to minimize thrombosis and mineral deposition. Biomimetic design strategies may involve the selection of scaffold materials, architectural features, cell types, or fabrication techniques, each targeting specific aspects of native valve structure and function.

### 3.1. Scaffold Materials

In both native and engineered environments, a reciprocal relationship exists between cells and the extracellular matrix (ECM); biomaterials influence cellular behavior through their chemical and mechanical properties, while cells actively remodel the surrounding microenvironment. Consequently, tissue-engineered heart valve (TEHV) scaffolds must simultaneously withstand cyclic hemodynamic loading and facilitate long-term biological integration. Current research focuses on two primary categories: decellularized extracellular matrix (dECM) and artificial polymeric scaffolds [3,4,6].

While dECM—derived from allogenic or xenogenic donors—provides high structural and compositional fidelity, its clinical utility is often hindered by donor scarcity and potential immunogenic risks. These limitations have prompted a shift toward synthetic and natural polymers, which offer a more scalable and tunable alternative. Polymeric scaffolds facilitate precise control over shape, porosity, and mechanical behavior, and can be further functionalized into “smart” biomaterials that direct specific cell adhesion and differentiation via bioactive ligands [3,4,6].

- dECM Scaffolds maintain native architecture but require rigorous decellularization to minimize immunogenicity, which can subsequently compromise mechanical integrity [3,4,6].
- Polymeric Scaffolds provide an essentially unlimited supply and controlled degradation kinetics, though they often require surface modifications to match the intrinsic bioactivity of native tissue [3,4,6].

#### 3.1.1. Decellularized ECM

Decellularized extracellular matrix (dECM) scaffolds offer high structural fidelity but face significant translational hurdles. While decellularization protocols were designed to reduce immunogenicity and improve host cell infiltration, xenogenic valves have encountered clinical failures due to inflammation, calcification, and poor recellularization. In contrast, allogenic valves demonstrate superior hemodynamic performance and cell survival, yet their application is constrained by donor scarcity [6,18,19,20,21,22,23,24,25,26,27].

To mitigate the mechanical deterioration often associated with decellularization, recent post-processing strategies, such as temporary polyvinyl alcohol (PVA) encapsulation, have successfully enhanced the tensile strength, handling, and suturability of porcine matrices without compromising cytocompatibility. For instance, Chen et al. [28] investigated the PVA as a dynamic encapsulation layer for decellularized porcine heart valves. They demonstrated that PVA-treated decellularized porcine heart valves exhibited significantly increased tensile strength and elastic modulus compared to untreated controls, effectively counteracting the typical mechanical deterioration caused by decellularization. The PVA layer also stabilized the valve structure during suturing procedures. After PVA removal, the modified valves retained improved mechanical integrity and supported robust adhesion and proliferation of human umbilical vein endothelial cells in vitro, confirming excellent cytocompatibility. These findings suggest that controlled post-processing approaches may mitigate some of the mechanical limitations associated with decellularization.

Despite these advancements, the success of dECM remains highly dependent on precise immunomodulation and remodeling control, motivating the development of more tunable synthetic alternatives [29].

#### 3.1.2. Synthetic Polymers

Synthetic polymers are widely used in TEHV scaffold fabrication due to their tunable mechanical properties and degradation kinetics. Common materials include polyglycol acid (PGA), polycaprolactone (PCL), polylactic acid (PLA), poly L-lactic acid (PLLA), and poly(glycerol sebacate) (PGS), all offering controlled performance under physiological loading conditions [3,4,13,28,30,31,32,33].

PGA and its copolymers are among the most established materials because of their high mechanical strength and predictable degradation profile, often combined with polymers such as P4HB or PLLA to better match tissue regeneration timelines [34]. PCL is biocompatible and easily processed, with mechanical properties adjustable through molecular weight and fabrication parameters [35]. PGS, an elastomeric polymer, degrades more rapidly (4–6 weeks) and allows modulation of mechanical behavior through curing conditions. Electrospun PGS:PCL composites have demonstrated anisotropic properties comparable to native valve tissue and supported robust cell growth in preclinical models [32].

PLA and PLLA exhibit slower hydrolysis due to more stable ester bonds, with degradation spanning 12–24 months or longer [34]. These materials are frequently combined with biological matrices to balance durability and remodeling capacity. For example, PLA-reinforced sandwich scaffolds have significantly enhanced tensile performance while maintaining endothelial compatibility [36].

Degradation kinetics vary substantially: PGA typically resorbs within months (often ~8 weeks in vivo), PGS within weeks, PLA/PLLA over 1–2 years, and PCL over 1–3 years under physiological conditions. Matching degradation rates to tissue regeneration remains essential in TEHV design [3].

Overall, synthetic polymers provide precise control over scaffold architecture and mechanics but often require biofunctionalization to enhance cell adhesion and long-term remodeling.

#### 3.1.3. Natural Polymers

Natural polymers, including fibrin, collagen, gelatin, hyaluronic acid (HA), chondroitin sulphate (CS), alginate, and polyhydroxyalkanoates such as P4HB, have been widely explored for TEHV scaffolding [3,4]. These biomaterials more closely mimic the extracellular matrix (ECM), providing a favorable microenvironment for cell adhesion, migration, and matrix deposition. However, due to limited mechanical robustness, natural polymers are most commonly used as hydrogels or cell carriers in hybrid systems combined with synthetic frameworks [3,4].

Fibrin is one of the most frequently applied natural polymers in TEHVs. Its degradation occurs through enzymatic cleavage by plasmin and matrix metalloproteinases (MMPs), and can be tuned by adjusting fibrinogen/thrombin concentrations or adding protease inhibitors [3,4]. Kreinin et al. investigated fibrin clot formation in prosthetic valves using a 3D-printed pulsatile aortic valve model and demonstrated that fibrin deposition strongly depended on valve orientation and local flow conditions, with increased clotting in low-shear recirculating regions, supported by complementary CFD analysis [37].

**Figure 1:**
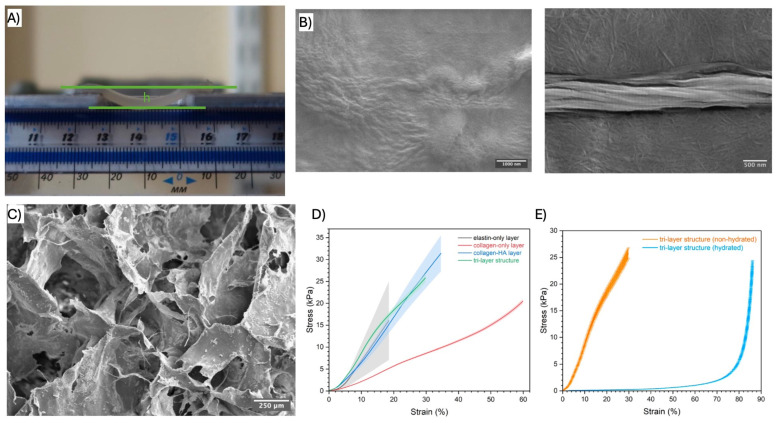
(**A**) Hydrated scaffold showing deflection measurements. (**B**) SEM images of the collagen suspension before (left) and after (right) fibrillogenesis. The left image lacks distinct fibers, whereas the right image reveals fibrillar atelocollagen in the background and the characteristic D-spacing pattern along the central fiber bundle formed post-incubation. (**C**) SEM image of crosslinked collagen-only scaffolds at 50× magnification. (**D**) Compressive stress–strain curves for the trilayer scaffold and its individual component layers in the non-hydrated state; shaded regions indicate standard deviation. (**E**) Compressive stress–strain curves for the trilayer scaffold comparing non-hydrated and hydrated states, with error bars representing standard deviation. Adapted from Ma et al. [38], Exploration of BioMat-X, 2024, licensed under CC BY 4.0.

Collagen, the primary structural component of the fibrosa layer in native valves, is highly cytocompatible and exhibits low antigenicity, making it an ideal candidate for TEHV scaffolds [3,4]. Ma et al. developed a biomimetic trilayer construct composed of atelocollagen, HA, and a fibrillar elastin gel to reproduce leaflet anisotropy (Figure 1). Their scaffold exhibited hydration-dependent nonlinear stress–strain behavior and physiologically relevant bending mechanics, closely resembling native valve tissue [38].

**Figure 2:**
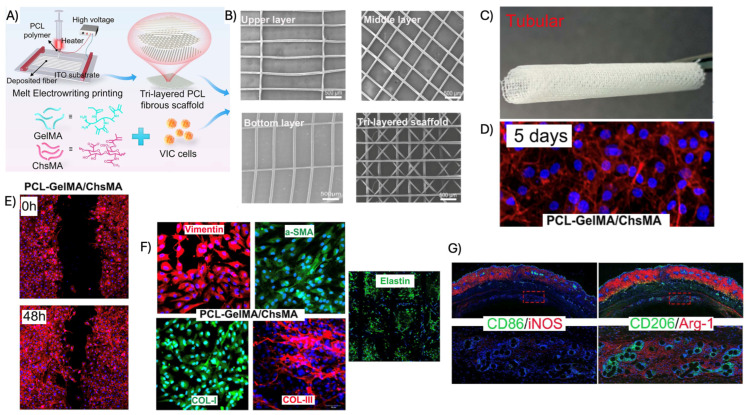
(**A**) Fabrication of a biomimetic heart valve tissue-engineered (HVTE) scaffold using MEW-enabled tri-layered PCL combined with 3D cell culture in bioactive hydrogels (GelMA/ChsMA). (**B**) Fiber organization in the tri-layered MEW-PCL scaffold: transversely aligned fibers on the top layer, diamond-shaped fibers in the middle, and vertically aligned fibers on the bottom; includes SEM image of the assembled scaffold. (**C**) Photograph of the tubular tri-layered MEW-PCL scaffold. (**D**) F-actin and DAPI staining of HUVECs cultured on scaffold surfaces after 5 days. (**E**) Scratch wound healing assay at 0 and 48 h. (**F**) Expression of Vimentin, α-SMA, COL-I, COL-III, and Elastin in VICs 3D-cultured within PCL-GelMA and PCL-GelMA/ChsMA scaffolds. (**G**) Representative immunofluorescence images after 4-week implantation, showing CD86/iNOS for M1 macrophages and CD206/Arg-1 for M2 macrophages. Red frame indicates the zoomed area displayed on the bottom. Adapted from Xu et al. [14], *Journal of Nanobiotechnology*, 2024, licensed under CC BY 4.0.

Gelatin, a denatured derivative of collagen, is commonly modified into gelatin methacrylate (GelMA), which can be UV-crosslinked into hydrogels with tunable properties. Xu et al. [14] combined melt-electrowritten anisotropic PCL scaffolds with GelMA/chitosan methacrylate hydrogels (Figure 2), producing composites that matched native leaflet mechanics, supported valvular interstitial cell growth, and improved hemocompatibility. In vivo implantation further showed reduced immune activation and suppressed calcification compared to PCL-only scaffolds.

Hyaluronic acid (HA), a glycosaminoglycan naturally present in valve ECM, regulates hydration and cell behavior [3,4]. Lei et al. demonstrated that incorporating HA into fibrin scaffolds altered microstructure and reduced leaflet retraction forces by ~25%, addressing a key failure mode associated with excessive myofibroblast contraction [39].

Overall, natural polymers offer superior biocompatibility and intrinsic cell-instructive properties compared to synthetic materials. However, their limited mechanical strength and often rapid degradation generally prevent their use as standalone valve scaffolds. As a result, natural polymers are most effectively incorporated into composite or hybrid designs, where they complement synthetic components to achieve an optimal balance of biological functionality, mechanical stability, and controlled degradation, more closely emulating native heart valve tissue. Scaffold material selection therefore requires careful consideration of both immediate structural demands and long-term regenerative potential. To support this comparison, Table 1 summarizes the key properties of the most prominent polymer systems investigated for TEHV applications.

#### 3.1.4. Hybrid Materials

Hybrid scaffolds that combine synthetic and natural polymers have emerged as a valid strategy in TEHV design, as they synergistically exploit the mechanical robustness and tunable degradation of synthetic frameworks with the bioactivity and cell-instructive properties of natural matrices. In these constructs, synthetic polymers such as PGA, PCL, or PLLA provide structural integrity and anisotropic reinforcement, while natural components such as collagen, fibrin, gelatin, or hyaluronic acid enhance cell adhesion, migration, and ECM remodeling [3,4].

Several studies highlight the advantages of this approach. Agnieszka et al. demonstrated that PGA/P4HB scaffolds enable tailored degradation while maintaining mechanical stability suitable for valve regeneration [6]. Similarly, Masoumi et al. reported electrospun PGS:PCL trilayer scaffolds achieving biomimetic anisotropy and functional leaflet motion in an ex vivo porcine model [32]. More recently, Xu et al. combined melt-electrowritten PCL architectures with GelMA-based hydrogels, producing composites that matched native leaflet mechanics while reducing immune activation and calcification in vivo [14].

### 3.2. Scaffold Architectural Design

Beyond material composition, scaffold architecture plays a decisive role in translating material properties into functional valve behavior. The architectural properties of scaffolds play a key role in mechanical properties, cellular attachment, growth, alignment and consequently construct functionality [6]. To facilitate physiological valve function, TEHVs aim to replicate the native tissue architecture, which is characterized by pronounced anisotropy and a multilayered organization. Fibrous and hydrogel scaffolds have been extensively employed to mimic the structural features of native heart valves, characterized by the fibrosa, ventricularis/atrialis and spongiosa layers which are enriched in collagen, glycosaminoglycans, and elastin fibers, respectively.

**Figure 3:**
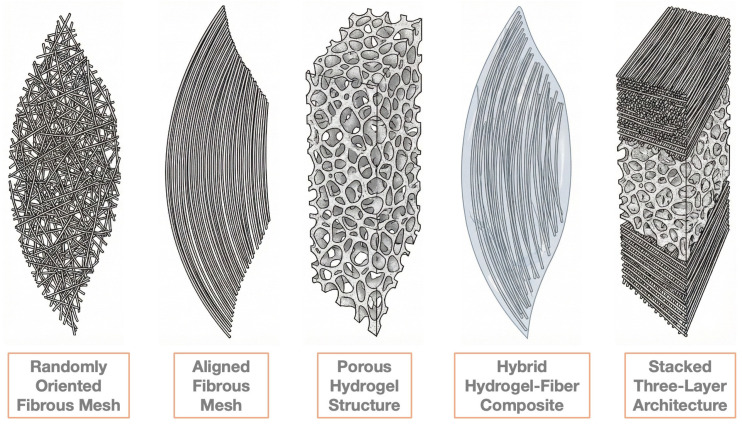
Schematic illustration of scaffold architectural designs impacting TEHV function. From left to right: randomly oriented fibrous meshes provide isotropic strength and suture retention but lack native anisotropy; aligned fibrous scaffolds mimic circumferential collagen organization to improve physiological anisotropic mechanics; porous hydrogel structures utilize natural polymers to create a hydrated niche for cell migration and extracellular matrix (ECM) secretion; hybrid hydrogel–fiber composites combine structural synthetic fibers for load-bearing durability with bioactive hydrogels to enhance hemocompatibility; biomimetic trilayered designs replicate native leaflet stratification (fibrosa, spongiosa, ventricularis/atrialis) to achieve nonlinear mechanical behavior and optimize cell–matrix interactions across different layers.

Importantly, scaffold morphological properties—such as fiber alignment, fiber diameter, pore size, and overall porosity—strongly influence cell–biomaterial interactions, mechanical performance, and degradation behavior [40]. As a result, scaffold architecture (Figure 3) represents a critical design variable in achieving biomimetic valve function.

#### 3.2.1. Fiber-Based Scaffolds

Fibrous scaffolds have been widely employed in TEHVs fabrication, using both randomly and directionally oriented fibers. Randomly oriented fibrous scaffolds are selected due to their relatively isotropic mechanical strength and ability to provide adequate suture retention strength in both circumferential and radial directions, which is critical for valve leaflet implantation [3]. Furthermore, the morphological characteristics of fibrous scaffolds, such as fiber diameter and pore size, can be tuned by adjusting polymer concentration and other fabrication parameters [3]. Recent studies demonstrated that randomly oriented fibrous scaffolds composed of PCL maintained stability under physiological loading conditions, while supporting cellular adhesion and growth [41]. However, despite these advantages, randomly oriented fiber architectures do not accurately reproduce the anisotropic structural and mechanical properties of native tissues. This limitation may lead to suboptimal cell alignment, altered mechanotransduction, and potentially adverse tissue remodeling over time [42].

To address these limitations, aligned fibrous scaffolds have been developed to more closely mimic the circumferentially alignment of collagen fibers found in the fibrosa layer of native heart valves, thereby reproducing anisotropic mechanical behavior [42]. Aligned fibrous architectures have also been combined in layered configuration to replicate the lamellar structure of native heart valve leaflets. For example, Gürbüz et al. constructed radially aligned PCL/PGS/polysulfone nanofibrous scaffolds through 3D printing and electrospinning. The resulting scaffolds mimicked the structural and anisotropic mechanical characteristics of the ventricularis layer, enhanced human umbilical vein endothelial cell (HUVEC) viability, and induced cell alignment demonstrating the potential of these composite scaffolds for TEHV applications [43].

#### 3.2.2. Hydrogel Structures

Hydrogel structures are often utilized in TEHVs as biocompatible layers, and occasionally as primary scaffolding materials. A critical characteristic of hydrogels is their network pore size, which significantly influences cell behavior, including proliferation, differentiation, morphology, and ECM secretion. This pore size can be tuned through variations in polymer concentration, molecular weight, or the degree of crosslinking [3]. Recent studies have demonstrated that hydrogels composed of biopolymers like gelatin and alginate can facilitate cell migration and enhance tissue regeneration when applied in TEHV constructs [44]. Moreover, the mechanical properties of hydrogel-based TEHVs can be modulated via biomechanical conditioning to achieve the mechanical stability essential for proper valve function and to mimic some mechanical aspects of native heart valve tissues [45]. In the absence of dynamic conditioning, however scaffolds made solely from hydrogels generally exhibit stiffness and ultimate tensile strength values that are significantly lower than those of native valve tissue, limiting their standalone applicability in TEHVs [44,45].

#### 3.2.3. Hybrid Hydrogel–Fiber Structures

To combine the favorable biological properties of hydrogels with the load-bearing capacity and anisotropy of fibrous scaffolds, hybrid hydrogel–fiber architectures have been increasingly adopted in TEHV design. This strategic combination facilitates the mimicry of both mechanical and biological properties of native heart valve tissue to a considerable extent, addressing limitations associated with either material class alone. Hybrid constructs facilitate the tuning of mechanical properties by modifying various parameters, such as fiber orientation, layer number, pore size, and hydrogel composition [3]. Robinson et al. reported that electrospun polyurethane meshes combined with hydrogel coatings yielded composite valve materials with adjustable mechanical behavior, reduced platelet and bacterial adhesion, decreased calcification, and hemodynamic performance comparable to clinical bioprosthetic valves, meeting ISO 5840-2:2021 standards [45]. These findings highlight the potential of hybrid architectures to achieve both mechanical robustness and biological functionality in TEHVs.

#### 3.2.4. Three-Layer Scaffolds

**Figure 4:**
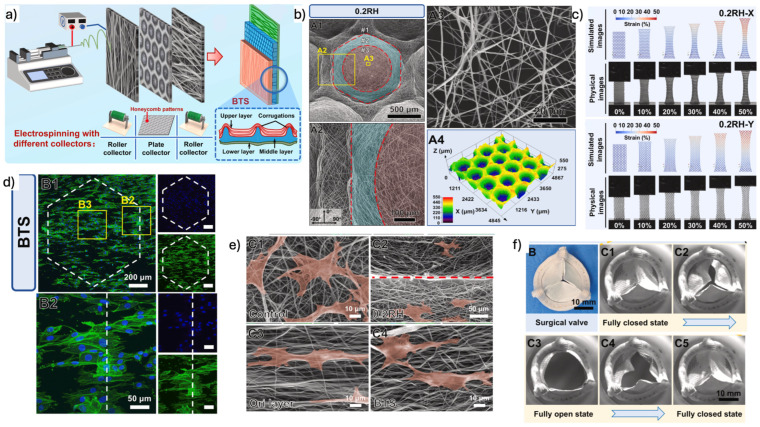
(**a**) Schematic of the fabrication of the upper, middle, and lower layers with distinct fiber morphologies via electrostatic spinning using different collectors, and the assembled biomimetic trilayer scaffold (BTS). (**b**) Top views of fibrous membranes and their distinct layers (#1–#3) with various honeycomb patterns (A1–A4: 0.2RH). (**c**) Finite element meshes for 0.2RH-X (hexagon diagonal) and 0.2RH-Y (hexagon opposite edges) showing displacement (mm) along the loading direction at strains from 0 to 50%, obtained via simulation. (**d**) Fluorescence images of A7r5 cells cultured on BTS fiber membranes for 3 days (green: cytoskeleton; blue: nuclei), B2 and B3 indicate border and central zooms respectively on the honeycomb pattern, only B2 is shown for simplified visualization. (**e**) SEM images of A7r5 cells (highlighted in red) on (C1) control, (C2) 0.2RH, (C3) Ori layer, and (C4) BTS fiber membranes. (**f**) Physiological hydrodynamic testing of the assembled pulmonary valve (B): valve opening and closing states throughout a heartbeat cycle (C1–C5). Adapted from Zhang et al. [47], *Chemical Engineering Journal*, 2025, licensed under CC BY 4.0.

To better replicate the complex architecture of native heart valve tissue, three-layer scaffold designs have been developed. In these constructs, TEHVs typically consist of two external anisotropic fibrous layers designed to mimic the fibrosa and ventricularis/atrialis, and a central more isotropic layer composed of glycosaminoglycans (GAGs) or natural polymer hydrogels to represent the spongiosa [3,46]. Recent advancements in this area include the work of Zhang et al. who fabricated bioinspired trilayer poly(ε-caprolactone) scaffolds using patterned electrospinning collectors to generate distinct layer-specific architectures (Figure 4). Their scaffolds reproduced native-like microstructural features, anisotropic mechanical behavior, and nonlinear stress–strain responses characteristic of valve leaflets. In vitro studies demonstrated cytocompatibility, guided cell alignment, and proliferation, while hydrodynamic testing confirmed effective orifice area and regurgitation rates compliant with ISO 5840-2 standards [47]. Collectively, these results underscore the relevance of trilayer scaffold architectures in approximating native valve mechanics and function.

### 3.3. Cell Types

The success of tissue-engineered heart valves (TEHVs) relies not only on mimicking the structural and mechanical properties of the native ECM but also on the proper selection of cell types for scaffold seeding. The selected cells must replicate the functional and structural roles of native heart valve cells while maintaining ECM synthesis, remodeling, mechanical adaptability, and signaling [4]. Among the various cellular candidates investigated for TEHV applications, valvular interstitial cells, valvular endothelial cells, and human umbilical vein endothelial cells have been most extensively studied.

Valvular interstitial cells (VICs) represent the predominant population within valve leaflets and are distributed throughout all layers of the tissue. They exhibit remarkable phenotypic heterogeneity, sharing characteristics with both fibroblasts and smooth muscle cells (SMCs) [4]. The ability of VICs to produce ECM proteins, including collagen, GAGs, and elastin, provides them with fibroblast-like traits. VICs can transition between a quiescent fibroblast-like phenotype and an activated myofibroblast phenotype, particularly in response to mechanical or biochemical stimuli [48]. This phenotypic plasticity is essential for normal valve growth, remodeling, and repair. However, sustained or excessive activation of VICs can lead to pathological remodeling, including fibrosis and calcification. Consequently, scaffold designs that accurately replicate the anisotropic and viscoelastic mechanical environment of native valve tissue play a crucial role in maintaining VICs in a physiological, quiescent state. Recent studies from De Morales et al. have demonstrated that VICs seeded on biomimetic scaffolds fabricated from decellularized ECM significantly enhance ECM remodeling and biocompatibility in TEHV constructs, supporting their effectiveness in mimicking native valve function [33]. These findings underscore the importance of matching scaffold mechanical and structural properties to native valve tissue to regulate VIC behavior effectively.

Valvular endothelial cells (VECs) line the blood-contacting surfaces of valve leaflets and play a pivotal role in maintaining homeostasis. They provide a non-thrombogenic barrier and actively regulate VIC behavior through paracrine signaling, thus modulating inflammation, thrombosis and remodeling processes [4]. VECs possess distinct morphologies and functions compared to vascular endothelial cells; for example, they position themselves perpendicular to blood flow, allowing them to withstand complex shear stress conditions. Additionally, VECs proliferate more rapidly and produce nitric oxide (NO), which aids in maintaining VIC quiescence [48]. Accordingly, scaffold architectures that support physiological shear stress exposure and appropriate endothelial alignment are critical for promoting functional endothelialization in TEHVs. Several studies have demonstrated that scaffolds engineered to favor VEC adhesion and alignment significantly improve hemocompatibility and long-term valve performance in vitro [48].

Human umbilical vein endothelial cells (HUVECs) are commonly leveraged in the development of TEHVs as they are easily isolated and cost-effective [4]. When seeded onto electrospun or otherwise fibrous scaffolds, HUVECs have been shown to form a continuous endothelial monolayer aligned with the flow direction, enhancing scaffold hemocompatibility and closely mimicking the anti-thrombogenic function of natural endothelium [4]. Although HUVECs do not fully recapitulate the phenotype of native VECs, they are frequently employed as a surrogate endothelial cell source for in vitro studies and early-stage validation of scaffold designs. Recent studies have reported that functionalization of bioprosthetic heart valves with HUVEC-derived extracellular vesicles or growth factor-rich coatings can enhance endothelialization, reduce thrombosis, and suppress calcification, further supporting the utility of HUVEC-based strategies in TEHV development [49].

In addition to utilizing native valve cells and HUVECs, several other cell types, including fibroblasts, have been proposed for TEHV development to enhance biomimetic outcomes. Fibroblasts are crucial components of connective tissue; they are spindle-shaped cells that contribute to ECM formation and maintain tissue integrity. They are the primary cells responsible for the development and repair of connective tissue, producing a highly ordered, collagen-rich matrix that facilitates force transmission and intricate tissue deformation [4]. Dermal fibroblasts have emerged as an alternative to VICs, offering advantages such as abundant ECM production and responsiveness to mechanical and biochemical stimuli. Dermal fibroblasts carry out multiple functions, including autocrine and paracrine signaling, proliferation, migration in response to chemotactic and mitogenic cues, and the synthesis and deposition of extracellular matrix. In a study by Syedain et al., dermal fibroblasts were seeded into decellularized heart valves using a bioreactor system, and the resulting tissue-engineered heart valves, tested in a pulse duplicator, exhibited structural properties like native valves. The use of dermal fibroblasts to generate a scaffold suitable for host cell recellularization provides a promising alternative to current tissue valve replacements [50].

Recent investigations have also provided mechanistic insights into fibroblast and VIC regulation. Batan et al. demonstrated that phosphatase and tensin homolog (PTEN) expression plays a critical role in regulating VIC activation in response to matrix stiffness. Their findings showed that PTEN overexpression preserved a quiescent fibroblast-like phenotype, whereas PTEN inhibition promoted myofibroblast activation, suggesting that pharmacological modulation of cell–matrix signaling pathways may represent a future strategy for controlling pathological remodeling in valve disease [51]. These findings highlight the importance of selecting appropriate cell types for TEHVs and underscore the need for integrating different cellular sources to achieve desired mechanical and biological properties for optimal heart valve function.

The selection of an appropriate cell source is a pivotal decision in TEHV design, balancing biological authenticity with practical constraints such as scalability and phenotypic stability. To facilitate a clearer comparison of current strategies, Table 2 summarizes the advantages and limitations of the most frequently utilized cell types, ranging from native valvular populations to cost-effective surrogates.

Beyond the selection of individual cell sources, co-culture strategies are increasingly recognized as essential for recapitulating the native cellular heterogeneity and paracrine signaling of heart valves. By spatially organizing multiple cell types—such as seeding VECs on the surface of a VIC-laden matrix—researchers can better simulate the physiological ‘crosstalk’ required for long-term homeostasis. For instance, Jesus De Morales et al. demonstrated that maturing iPSC-derived mesenchymal stem cells into VIC-like cells within a structured environment significantly promotes healthy ECM markers. Furthermore, modern hybrid approaches, such as the sandwich structures described by Zhang et al. provide the necessary architectural cues to support these heterogeneous populations, ultimately reducing pathological activation and improving hemodynamic performance [33,47].

### 3.4. Fabrication Techniques

Various scaffolding techniques have been utilized to fabricate TEHVs, including molding, electrospinning, jet-spinning, melt electrowriting, textile methods, and photolithography. Often, these methods are combined, such as using electrospinning in conjunction with molding to fabricate hybrid scaffolds that can better replicate the complex structure and mechanical properties of native heart valves [3].

#### 3.4.1. Molding

Molding has emerged as a widely used technique for creating TEHV scaffolds due to its high throughput and capacity to produce complex valve-relevant geometries [3]. This approach typically involves the use of molds, with tubular or more complex shapes—such as bi- or trilayered structures—into which various materials, primarily hydrogels, can be injected [3]. Hydrogels molded into tubular structures can be transformed into bi- or trilayered valves, either by suturing concentric layers together or using a supporting frame [52]. When hydrogels are directly molded into bi- or trilayered structures, the resulting geometry depends on the mold design, typically generated via computer-aided manufacturing methods [52]. Recent studies have demonstrated that GelMA- and Poly(ethylene glycol) diacrylate (PEGDA)-based hydrogels can maintain structural integrity and allow for adequate cell infiltration when molded into valve-like geometries [33]. However, it is essential to note that scaffolds fabricated solely from cell-laden hydrogels without biomechanical conditioning often exhibit inadequate mechanical strength and limited anisotropy, limiting their ability to replicate the functionality of native heart valve tissues [53]. To address this limitation, molding is frequently combined with other fabrication methods, such as electrospinning, to enhance mechanical performance [53].

#### 3.4.2. Electrospinning, Jet-Spinning and MEW

Electrospinning is one of the most employed techniques for producing fibrous scaffold using various natural and synthetic polymers, such as collagen, P4HB, PCL-PLLA, PCL, PGS, polyurethane (PU), and PLLA [3]. This technique generates precursor sheets or tubes that can later be transformed into bi- or trilayered structures using suturing or origami methods, or it may directly create the desired structures thanks to specialized collectors [52]. Electrospinning generates micro- to nanoscale fibers through the application of a high-voltage electric field, which draws a charged polymer jet from a spinneret toward a grounded collector. Electrospun fibrous scaffolds closely resemble the native fibrous microarchitecture of native valve ECM, making it particularly attractive for biomimetic scaffold design [53]. The orientation and deposition of fibers can be controlled by adjusting the shape and movement of the collector, allowing for the development of scaffolds with anisotropic structures and mechanical properties that mimic those of native valve tissues [52]. Adjustments in parameters such as voltage, flow rate, and polymer concentration can facilitate control over fiber diameter, morphology, and alignment. These factors significantly impact the mechanical behavior and biological responses (e.g., cellularization) of the final TEHV [33]. Moreover, electrospun scaffolds feature a high surface-to-volume ratio, enhancing cellularization both in vitro and in vivo [4]. However, cellularization in electrospun constructs tends to occur primarily on the surface, with limited infiltration due to the dense fibrous structures. Additionally, the electrospinning method has limitations concerning the control of the architecture, diameter, and orientation of fibers making scaffolds susceptible to unwanted variations caused by fluctuations in the electric field and polymer viscosity [53].

Jet spinning represents an alternative fiber fabrication technique that relies on high-speed rotational forces rather than electric fields. This method is generally more robust to environmental fluctuations and allows improved control over fiber deposition compared to electrospinning [3]. However, jet spinning is typically limited to simpler fiber architectures and offers less flexibility in generating complex three-dimensional geometries required for TEHVs.

Melt electrowriting (MEW) combines 3D printing and melt electrospinning to offer superior control over fiber morphology and has gained increasing attention in generating fibrous scaffolds, mainly using PCL, for TEHV applications [3]. Like electrospinning and jet spinning, MEW allows for the deposition of fibers in various geometries, including cylindrical, planar, or more anatomically relevant structures [3]. Recent studies illustrate that MEW has been effective in creating scaffolds with a crimped fiber architecture, replicating the nonlinear stress–strain behavior typical of native valve tissue [3]. Despite these advantages, MEW suffers from relatively low throughput and sensitivity to processing conditions, posing challenges for large-scale manufacturing. Additionally, issues related to charge accumulation and limited microporosity might constrain cell infiltration [53,54,55].

#### 3.4.3. Textile Technologies

**Figure 5:**
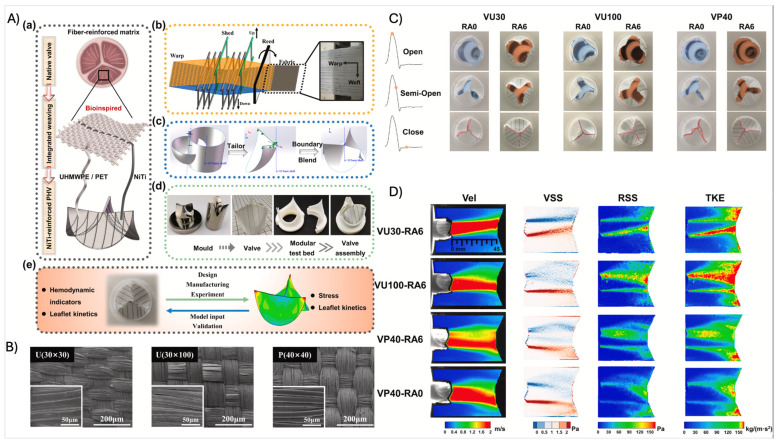
(**A**) Biomimetic design and fabrication of NiTi-TexVal prototype. (a) Biomimetic design strategy; (b) Fabrication of integrated hybrid micro-tube; (c) Computer modeling of trileaflet polymeric valves; (d) Shaping and assembly of valve prototypes; (e) Integration of in vitro experimental research and numerical simulation to advance the field of heart valve prosthesis. (**B**) Characterization of leaflet materials. (**C**) Hemodynamic indicators and morphology of different valve designs. Images depict representative pictures of different valve designs at critical phases, including fully open during peak systole, semi-open during end systole, and fully closed during early diastole. (**D**) Flow field characteristics of different valve designs: contours of velocity, VSS, RSS, and TKE at peak systole downstream of the valves (about 45 mm). Adapted from Chen et al. [56], *Composites Part B*, 2023, licensed under CC BY 4.0.

Textile technologies, such as weaving or knitting, utilize polymeric fibers produced by methods such as electrospinning to fabricate yarns, which are then transformed into textiles. These techniques have been employed for the development of tissue sheets or tubular structures, primarily composed of synthetic polymers such as PCL, poly(ethylene terephthalate), and poly(acrylonitrile) [3]. The scaffolds generated through this method are characterized by tunable mechanical and degradation properties, mechanical anisotropy, and structural stability. Consequently, textiles have also been employed as supporting backbones to reinforce hydrogel structures combining mechanical stability with biological functionality. For example, woven PCL scaffolds have been shown to significantly enhance mechanical integrity and cell attachment when integrated into TEHV constructs [3]. Chen et al. further demonstrated that reinforcing polymeric heart valve matrices with radially oriented NiTi wires, using textile technologies, improved leaflet kinematics, reduced stress concentrations, and resulted in favorable hemodynamic performance, highlighting the potential of textile-based hybrid designs for durable valve replacements (Figure 5) [56].

#### 3.4.4. Cell-Free Approach

While both cellularized and cell-free approaches aim for functional restoration, they offer distinct trade-offs in clinical and industrial contexts.

Cell-free (in situ) approaches are currently the preferred strategy for large-scale production and emergency clinical use due to their superior manufacturability and “off-the-shelf” availability. By eliminating the need for complex, patient-specific cell harvest and expansion, acellular scaffolds—particularly synthetic polymeric or decellularized xenogenic matrices—reduce the risk of phenotypic drift and the high costs associated with prolonged in vitro culture [3,6,10,39].

In contrast, cellularized approaches (in vitro TE) remain the gold standard for scenarios requiring immediate biological integration and high-rate ECM turnover, such as in pediatric patients where somatic growth is a primary requirement. However, cellularized constructs face significant safety challenges, including the risk of overactive remodeling and myofibroblast-mediated calcification. From a production standpoint, the scalability of cellularized TEHVs is limited by batch-to-batch variability and the lack of standardized maturation protocols. Consequently, while cellularized valves offer higher initial biological potential, cell-free scaffolds functionalized with bioactive cues represent a more translatable middle ground for widespread clinical adoption [3,6,10,39].

#### 3.4.5. Comparison of Fabrication Technologies

Advanced techniques, such as melt electrowriting (MEW) and nanotechnology-based approaches, offer unmatched design flexibility, but their translation to clinical-scale manufacturing requires careful consideration of feasibility and cost. MEW provides excellent reproducibility for creating anisotropic fibrous architectures [3], yet the process remains relatively slow, which may limit high-volume production. Nanotechnology strategies, including targeted drug delivery systems, enable precise functional control [56] but bring added regulatory complexity and potential challenges in maintaining batch-to-batch consistency at scale. In contrast, acellular scaffolds produced via high-speed jet spinning [39,57] are cost-effective, easier to sterilize, and suitable for “off-the-shelf” use, although they lack the immediate biological complexity of bioprinted, cell-laden constructs.

Many fabrication techniques also allow tuning of scaffold porosity, a key parameter for TEHV performance. Molding generates complex 3D hydrogel structures, with porosity largely determined by the hydrogel formulation; typical pore sizes range from 10 to 100 µm, sufficient for cell infiltration but limited by mechanical constraints [3]. Electrospinning produces fibrous scaffolds with diameters from tens of nanometers up to several micrometers, resulting in small pores (<10 µm) that can hinder cell infiltration unless modified with porogens or patterned collectors [3,52,53]. Jet-spinning, which relies on high-speed rotation rather than electric fields, improves deposition consistency and robustness but typically produces simpler 3D architectures [3,52,53]. MEW, combining 3D printing with melt electrospinning, allows precise fiber placement and generates well-defined pores (100–300 µm) that support controlled cell behavior and tissue development. Unlike random electrospun networks, MEW scaffolds offer tunable architecture and anisotropy, closely mimicking native heart valve extracellular matrix while maintaining reproducible pore structures [3]. Finally, textile techniques, such as weaving or knitting, produce polymeric yarns with adjustable inter-fiber spacing, achieving pores of 100–500 µm that enhance mass transport and cellularization [56]. Together, these parameters can be optimized to support cell infiltration, ECM deposition, and the mechanical performance of the final construct.

**Figure 6:**
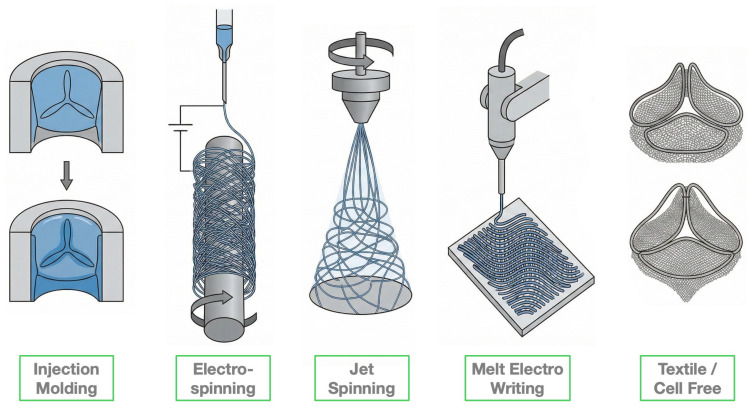
Overview of primary biofabrication techniques for TEHV scaffolds. From left to right: molding facilitates high-throughput generation of anatomical hydrogel geometries; electrospinning utilizes high-voltage fields to create fibrous meshes mimicking ECM microarchitecture; jet spinning (Rotary Jet Spinning) uses centrifugal force for scalable fiber fabrication; melt electrowriting (MEW) enables precise, computer-controlled deposition of microfibers for defined anisotropic patterns; hybrid/textile assembly integrates reinforced backbones (e.g., woven nitinol or polymeric fabrics) with hydrogels or electrospun layers for enhanced mechanical durability.

Ultimately, the clinical viability of TEHVs will likely depend on hybrid manufacturing strategies that combine the high-resolution precision of MEW with the speed and cost-efficiency of traditional textile or electrospinning methods. Effective scaffold design emerges from the coordinated integration of material composition, architectural organization, cellular interactions, and fabrication techniques, rather than from any single design element alone (Figure 6).

Summarizing, biomimetic scaffold design for TEHVs requires a careful balance of material properties, architectural features, and cellular components to replicate the structure and function of native heart valves. Decellularized ECM offers unmatched structural fidelity but is limited by donor availability and potential immunogenicity, whereas synthetic polymers provide tunable mechanical properties, degradation kinetics, and architecture, allowing precise control over scaffold porosity and anisotropy. Natural polymers contribute intrinsic bioactivity, promoting cell adhesion, migration, and ECM deposition, and are most effective when incorporated into hybrid constructs with synthetic frameworks to achieve both biological functionality and mechanical robustness. Scaffold architecture—from fibrous and hydrogel structures to hybrid hydrogel–fiber and trilayered designs—plays a pivotal role in guiding cellular organization, mechanical behavior, and tissue remodeling. The selection and spatial organization of cell types, including VICs, VECs, HUVECs, and fibroblasts, further modulate ECM synthesis, endothelialization, and long-term valve performance, with co-culture strategies enabling physiologically relevant paracrine signaling. Finally, fabrication techniques such as molding, electrospinning, jet-spinning, melt electrowriting, and textile methods provide complementary approaches to tailor scaffold geometry, pore size, and anisotropy, supporting both cellularized and cell-free strategies. Taken together, these integrated design considerations highlight the importance of a multiscale, multidisciplinary approach in developing TEHVs capable of recapitulating native valve mechanics, biological cues, and long-term functionality.

## 4. Biological Cues

Beyond structural and mechanical considerations, effective TEHV scaffolds must actively regulate biological interactions with the host environment, motivating the integration of bioactive cues discussed below. The ability of THEVs to grow and remodel upon implantation is strongly influenced by the host immune response. By incorporating bioactive factors into the implanted valve, it is possible to promote the recruitment of specific cell populations and modulate local cellular function [57]. In this context, biomimetic design plays a central role by reproducing the biochemical and structural cues naturally found in native heart valves. Scaffolds can effectively manage host–tissue interactions, steering them towards regeneration rather than fibrosis. Recent studies have further demonstrated that microenvironmental conditions, including biochemical gradients and biomaterial stiffness, strongly influence cell recruitment, differentiation, and functional maturation [53]. For instance, Cheng et al. showed that the maturation trajectory of pluripotent stem cell-derived cardiomyocytes is highly dependent on microenvironmental cues, highlighting the broader implications of biophysical and biochemical signaling in cardiac tissue engineering [58]. Accordingly, biological cues are increasingly integrated into TEHV designs to recapitulate key aspects of native valve biology and support constructive tissue remodeling. Antithrombotic coatings, immunomodulatory molecules, cell-interactive ligands, and drug delivery systems, that can be employed to emulate physiological processes and recreate a regenerative microenvironment, will be discussed in the subsequent sections.

### 4.1. Antithrombosis and Anti-Calcification Strategies

Despite advancements in biomaterial development to reduce hemolytic, toxic, and immunological reactions, thrombotic and thromboembolic, complications related to cardiovascular implants remain a major clinical concern. Upon contact with blood, a thin monolayer of plasma proteins quickly covers the surface of the implanted scaffold. The composition and conformation of these proteins depend on the surface chemistry of the biomaterial including charge, roughness, and porosity. These surface properties influence platelet adhesion and activation thereby affecting thrombus formation [6,59]. In physiological valves, the endothelial layer maintains hemostasis by presenting a surface that resists nonspecific protein absorption and prevents platelet activation [6]. Consequently, a major objective of biomimetic TEHV design is to reproduce these antithrombotic properties during the early post-implantation period.

Heparin functionalization is among the most widely adopted strategies to mimic the anticoagulant activity of endothelial cells. Heparin-coated scaffolds inhibit platelet aggregation and thrombin activity while also helping to mitigate calcification, modulating macrophage activity and reducing local inflammation [60,61,62]. Recent research has demonstrated the effectiveness of heparin-functionalized scaffolds in reducing thrombus formation in vivo; for example, Hu et al. reported that biological valve leaflets modified with heparin-loaded hydrogels exhibited enhanced hemocompatibility, improved endothelialization, reduced immune response, and strong anti-calcification performance, compared to conventional glutaraldehyde-treated tissues [63].

### 4.2. Immunomodulatory Cues

Following implantation, porous scaffolds experience early infiltration by circulating immuno cells, which release cytokines and growth factors that regulate cell recruitment, inflammation and tissue remodeling. Among these immune cells, macrophages play an essential role in resolving inflammation due to their ability to transition from a pro-inflammatory state (M1-like) to a reparative phenotype (M2-like) [6]. Rather than suppressing immune activation, modern biomimetic strategies aim to guide macrophage polarization toward pro-regenerative phenotypes, mirroring physiological wound healing processes.

Cytokines such as monocyte chemoattractant protein-1 (MCP-1) and stromal cell-derived factor 1α (SDF-1α) are illustrative examples of biomimetic signaling molecules that convert immune activation into regenerative processes. MCP-1 is known to promote the recruitment of circulating inflammatory cells, facilitating rapid and homogeneous infiltration of matrix by blood-derived cells [6,64]. Recent studies indicate that the release of MCP-1 from scaffold materials can enhance cell migration and retention, thereby boosting the regenerative capacity of implanted valves [6,65,66]. Similarly, SDF-1α has proven crucial in recruiting blood-derived tissue-producing progenitor cells and controlling valve cell phenotype. Its incorporation into TEHV architectures has been associated with improved endothelialization and ECM remodeling [6].

In addition to soluble factors, scaffold chemistry itself can exert immunomodulatory effects. Modifications using oxidized hyaluronic acid, peptide-functionalized surfaces, or ROS-responsive hydrogels have been shown to induce macrophage polarization toward M2 phenotypes, improve hemocompatibility, and reduce calcification in vivo [6]. These findings highlight the potential of embedding immunomodulatory function directly into scaffold design rather than relying solely on exogenous factor delivery.

### 4.3. Cell Recruitment, Adhesion and Differentiation

In native heart valves, cellular behavior is regulated by highly specific cell–matrix interactions mediated by integrins, adhesion ligands, and growth factor gradients. Biomimetic TEHV design thus focuses on recreating these interactions by functionalizing scaffold surfaces with bioactive molecules that regulate cell recruitment, adhesion, and differentiation [6]. Antibody-based strategies, such as functionalization with anti-CD34 or anti-CD133 antibodies, have been employed to capture circulating progenitor cells and accelerate endothelialization. While these approaches can enhance early cell attachment, they often lack specificity and may recruit heterogeneous cell populations [67,68,69]. In contrast, peptide-based ligands derived from extracellular matrix proteins offer more selective control over cell adhesion. The RGD peptide promotes integrin-mediated attachment of multiple cell types, whereas the REDV peptide preferentially binds endothelial progenitor cells, supporting targeted endothelialization [70,71]. Liu et al. demonstrated that decellularized porcine pericardium functionalized with ulvan, REDV, and Vascular Endothelial(VE)-cadherin antibodies significantly enhanced endothelialization while reducing thrombogenicity, inflammation, and calcification, both in vitro and in vivo, highlighting the regenerative potential of multifunctional surface modifications [70].

Growth factor incorporation represents a complementary strategy for regulating cell fate. Vascular endothelial growth factor (VEGF), growth differentiation factor 11 (GDF11), and nitric oxide (NO)-releasing systems have been shown to promote endothelialization, suppress platelet activation, and modulate inflammatory signaling [72,73]. GDF11, a Transforming Growth factor (TGF)-β superfamily member, promotes endothelial progenitor cell (EPC) proliferation, migration, survival, and angiogenic activity via TGF-β/Smad signaling, and has recently been implicated in EPC recruitment in ischemic models. Its integration into biomaterials represents a novel strategy for TEHVs. In this context, scaffolds incorporating copper-modified GDF11 enabled controlled NO release, enhancing EPC recruitment, hemocompatibility, and endothelialization while supporting a pro-vascular microenvironment [72,73].

More broadly, growth factor incorporation within biomaterials is an effective approach to modulate the local cellular niche and mimic physiological signaling. VEGF enhances endothelial cell migration, proliferation, and progenitor recruitment, while TGF-β1 regulates ECM synthesis and interstitial cell differentiation. Controlled immobilization, often via heparin-based strategies, ensures sustained bioactivity and spatiotemporally regulated release, thereby supporting functional tissue regeneration [6,54,74].

However, it is important to underline that reproducing these cues in a biomimetic manner requires precise spatial and temporal control to prevent excessive exposure and unintended effects.

### 4.4. Drug Loaded Scaffolds

A further evolution in biomimetic TEHV design involves the incorporation of drug delivery systems that locally reproduce the paracrine signaling environment of native tissue. Drug-loaded scaffolds can deliver anti-inflammatory, anti-thrombotic, or regenerative molecules in a controlled way, thereby mimicking the endogenous release of bioactive mediators following tissue injury [57,75]. However, physiological healing is a temporally dynamic process involving sequential signaling events. Consequently, biomimetic drug delivery systems must be engineered to provide spatiotemporal control over release kinetics, preventing the “burst release” phenomenon that often leads to rapid clearance and off-target effects. To achieve this, bioactive agents can be physically entrapped within scaffold fibers, chemically conjugated to polymer backbones, or encapsulated within nano- and micro-carriers dispersed within the scaffold matrix [4,6].

Recent approaches in TEHV design extend beyond growth factor delivery to include pharmacological agents that modulate cell phenotype and immune responses. For example, Simvastatin-loaded scaffolds have been shown to enhance cardiomyoblast proliferation and adhesion, while C-type natriuretic peptide (CNP) delivery suppresses pathological activation of valvular interstitial cells, preserving leaflet geometry and preventing stenosis [76,77].

Immunomodulation has emerged as a critical strategy, with controlled release of cytokines such as MCP-1 and SDF-1α guiding macrophage polarization from pro-inflammatory M1 to reparative M2 phenotypes. This approach reduces fibrotic encapsulation and promotes constructive tissue remodeling and endothelialization [78,79].

Biocompatible polymers and injectable hydrogels further support controlled drug release and tissue integration. Materials such as poly(ε-caprolactone) enhance growth factor retention, while chitosan–fucoidan hydrogels delivering FGF-2 improve neovascularization and scaffold integration [34,80]. In parallel, incorporation of anti-thrombotic agents, including nitric oxide-releasing systems, mitigates platelet activation and inflammatory responses, improving scaffold hemocompatibility and durability. Multifunctional hybrid scaffolds combining antithrombotic, immunomodulatory, and anti-calcific properties have demonstrated superior endothelialization and reduced calcification in vivo [73,81,82].

Nanoparticle-based delivery systems further expand TEHV functionality by enabling targeted and sustained release of therapeutic agents. Recent studies show that nanoparticles can modulate drug pharmacokinetics and reduce off-target toxicity while supporting endothelialization and suppressing calcification, for example through the co-delivery of RunX2-siRNA and VEGF [83,84,85].

**Figure 7:**
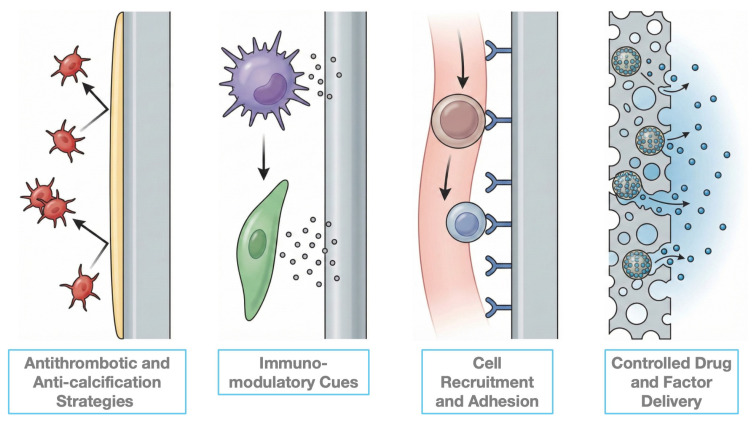
Strategies for biological functionalization of TEHV scaffolds to regulate host–tissue interactions. From left to right: antithrombotic surface modification (e.g., heparin coating) inhibits platelet activation and adhesion to mimic endothelial thromboresistance; Immunomodulatory signaling utilizes bioactive cues to guide macrophage polarization from pro-inflammatory (purple, spiky) to reparative (green, elongated) phenotypes; selective cell recruitment employs surface ligands (e.g., RGD or REDV peptides) or antibodies to capture circulating progenitor cells; controlled drug delivery systems, such as embedded nanoparticles, provide spatiotemporal release of growth factors (e.g., VEGF, GDF11) to promote endothelialization and remodel the microenvironment.

Overall, the integration of controlled drug delivery systems within TEHV scaffolds enables localized, biomimetic modulation of the cellular microenvironment, enhancing regeneration while limiting thrombosis, inflammation, and calcification, and represents a promising direction for next-generation heart valve engineering. Among the many biological cues explored (Figure 7), immunomodulation—particularly macrophage polarization—has emerged as a dominant determinant of constructive remodeling in vivo.

## 5. Biomimetic Bioreactors and Conditioning Systems

While scaffold design and biological cues define the regenerative potential of TEHVs, functional maturation depends on exposure to physiological environments. Heart valve disease continues to drive the clinical use of mechanical and bioprosthetic valve replacements; however, both options face limitations such as thrombogenicity, poor longevity, and a complete lack of growth potential, which is particularly problematic for pediatric patients. TEHVs have therefore emerged as a promising alternative, aiming to provide living, adaptable replacements capable of remodeling over time.

In this context, a bioreactor can be defined as a controlled in vitro culture device that maintains engineered tissues under regulated conditions, ensuring adequate nutrient delivery, waste removal, and environmental control. Beyond basic culture support, bioreactors are often coupled with conditioning systems, meaning the application of defined mechanical and biochemical stimuli—such as pulsatile flow, cyclic strain, and shear stress—to mimic the native valve environment and promote tissue maturation.

Central to TEHV development are therefore bioreactors and conditioning platforms designed to replicate physiological environments and deliver the cues necessary for functional tissue development. More importantly, these systems act as mechanobiological training tools, accelerating extracellular matrix (ECM) deposition, anisotropic fiber organization, and endothelial maturation prior to implantation [85,86].

Modern bioreactors are not simple culture chambers, but aim to reproduce key aspects of native valve biomechanics to guide tissue development toward functional competence. By tuning parameters such as strain amplitude, flexural frequency, and pulsatile shear stress, conditioning protocols can be optimized to improve leaflet strength, reduce maladaptive remodeling, and enhance long-term durability [87,88].

### 5.1. Mechanical Conditioning Platforms

**Figure 8:**
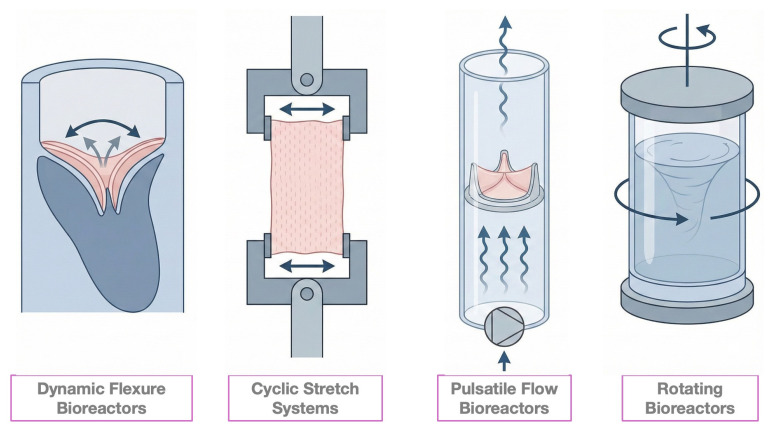
Schematic representation of mechanical conditioning platforms used for TEHV maturation in vitro with arrows representing the direction of applied stimuli. From left to right: Dynamic flexure bioreactors simulate repetitive leaflet bending during the cardiac cycle to promote collagen organization and bending stiffness; Cyclic stretch systems apply controlled tensile loading to stimulate extracellular matrix synthesis and enhance tensile strength; Pulsatile flow bioreactors recreate physiological hemodynamic conditions, including shear stress, to promote functional endothelialization; Rotating bioreactors utilize dynamic culture primarily to enhance mass transport and nutrient distribution throughout thick tissue constructs.

Several classes of bioreactors have been developed to mimic specific mechanical stimuli experienced by native heart valves (Figure 8), including dynamic flexure, cyclic stretch, pulsatile flow, and rotating bioreactor systems. Each platform targets distinct components of the valvular mechanical environment and contributes to tissue maturation.

- **Dynamic Flexure Bioreactors** simulate the repetitive bending experienced by heart valves during the cardiac cycle. By subjecting scaffolds to controlled flexural deformation, they help promote the alignment of cells and matrix deposition, which are critical for the function of natural heart valves [86]. This flexural loading is particularly relevant for establishing leaflet-like collagen architecture and improving bending stiffness [86].
- **Cyclic Stretch Bioreactors** apply intermittent stretching to engineered tissues, replicating the dynamic mechanical loading that native heart valves undergo. The application of cyclic stretch has been shown to enhance cell proliferation and matrix production, facilitating the creation of stronger and more resilient tissues. Therefore, stretching parameters can be tuned to optimize tissue growth and functionality [87]. In TEHVs, cyclic strain is also a key driver of VIC differentiation and tensile reinforcement of the developing leaflet [88].
- **Pulsatile Flow Bioreactors** simulate the hemodynamic conditions of the cardiovascular system. They create a pulsatile flow, like blood flow, which encourages endothelialization and the formation of a vascular network within the graft. By controlling the flow rate, pulsatile frequency, and pressure, these bioreactors can support realistic flow conditions that promote the development of layered tissues characteristic of native heart valves [87]. Such shear-mediated conditioning is essential for generating a functional endothelial monolayer and reducing thrombogenic risk post-implantation [89,90].
- **Rotating Bioreactors** use centrifugal forces to enhance mass transport and nutrient distribution within the scaffold. Although less specific to valve mechanics, these systems are particularly useful to improve cell distribution and oxygenation, ensuring that the cells embedded in the scaffold receive adequate environmental support to proliferate and secrete extracellular matrix components necessary for tissue formation [86].

### 5.2. Advantages of Dynamic Conditioning

Beyond conventional mechanical stimulation, dynamic conditioning strategies are increasingly being refined to better reproduce the coupled biomechanical environment of native valve leaflets. In vivo, heart valves are exposed simultaneously to cyclic flexure, tensile stretch, and complex pulsatile shear stress, and TEHV maturation depends on integrating these stimuli rather than applying them in isolation [85,87]. Consequently, next-generation bioreactors aim to deliver multi-axial loading regimes that more accurately guide tissue remodeling toward physiologic structure–function relationships.

Dynamic conditioning has been shown to enhance collagen deposition, promote anisotropic ECM organization, and support endothelial stability, all of which are essential for achieving leaflet durability and hemocompatibility. Importantly, conditioning protocols can be tuned to avoid maladaptive responses such as excessive myofibroblast activation, tissue compaction, or premature stiffening, which remain key failure modes in TEHV development [88,90]. For instance, Dumont et al. designed a novel pulsatile bioreactor that allowed for real-time monitoring of tissue responses under conditions that precisely replicated hemodynamic forces experienced within the cardiovascular system [91]. Their findings confirmed that pulsatile flow significantly enhances endothelialization of valvular tissues, effectively reducing the risk of thrombosis—a critical challenge in valve replacement therapies. The endothelial cell coverage observed under dynamic conditions was superior compared to static cultures, reinforcing the importance of mechanical stimulation in achieving a functional endothelial layer capable of promoting patency and functionality post-implantation.

### 5.3. Emerging Condition Strategies

Beyond conventional mechanical stimulation, recent studies have explored the integration of additional physical cues to further enhance tissue maturation. Gabetti et al. explored the effects of electrical stimulation in conjunction with pulsatile flow on the maturation of engineered cardiac tissues, revealing synergistic effects that collectively enhanced cellular alignment, proliferation, and ECM deposition [92]. Although primarily explored in myocardial models, such approaches highlight the potential of multimodal conditioning strategies for future TEHV applications.

Advances in bioreactor design have also enabled real-time monitoring and adaptive conditioning. Ongoing research by Voß et al. emphasizes the need for sensory systems within bioreactors that can detect and adapt to the real-time conditions of the implants, tailoring the mechanical conditioning to enhance patient-specific TEHV maturation processes [93]. This adaptability can significantly improve outcomes, particularly for pediatric patients who require growth potential in engineered valves due to their continuous development [94].

It has been observed that Magnetic Resonance Imaging (MRI) and ultrasound-compatible bioreactors allow non-invasive longitudinal assessment of valve motion, ECM formation, and mechanical performance under dynamic loading [95]. A metal-free bioreactor newly described by Mohapatra et al., compatible with high-field MRI (7T) and ultrasound imaging, enabled non-invasive, longitudinal monitoring of TEHV maturation under physiological dynamic loading (pressure, flow, cyclic motion). In experiments using textile-based biohybrid valves, conditioning in this bioreactor resulted in abundant ECM production, including collagen and α-smooth muscle actin, and formation of a coherent endothelial monolayer across the valve cusps [95]. This integration of real-time imaging with dynamic mechanical conditioning marks a critical step toward reproducible, controlled preclinical maturation protocols and potentially earlier indicators of in vivo performance. Such feedback-controlled systems may reduce batch-to-batch variability and accelerate the standardization required for clinical translation [85,90].

### 5.4. Integration with Computational Modeling

Computational modeling is increasingly integrated with experimental bioreactor platforms to improve the design, control, and predictive understanding of TEHV maturation. While bioreactors provide the physical stimuli required for tissue development, in silico models offer a complementary framework to quantify local mechanical environments, optimize culture parameters, and anticipate biological outcomes that are difficult to measure experimentally. In particular, computational tools can be used to predict scaffold deformation, flow-induced shear stress distributions, cell viability, and long-term tissue remodeling under different conditioning regimens, thereby reducing reliance on empirical trial-and-error approaches.

Recent finite-element frameworks, such as the model proposed by Sesa et al., enable the prediction of collagen deposition, anisotropic ECM remodeling, and valve deformation under physiologically relevant mechanical loading [96]. Such approaches provide powerful guidance for optimizing scaffold geometry, tailoring conditioning protocols, and improving pre-implantation validation. Moreover, computational studies can incorporate key design variables—including scaffold architecture, degradation kinetics, fiber orientation, and dynamic conditioning—to estimate long-term mechanical performance and maturation trajectories. Recent reviews highlight that properly engineered polyester-based scaffolds (e.g., PCL) may remain structurally stable for up to two years in vivo, providing a sufficient temporal window for endogenous tissue formation and integration [97].

Overall, the combination of biomimetic bioreactors and computational modeling strengthens TEHV development by enabling a more rational and reproducible maturation strategy. By capturing the interplay between hemodynamic forces, cyclic deformation, and scaffold–cell interactions, modeling enhances control over ECM deposition, fiber alignment, endothelialization, and differentiation. Static culture alone is no longer defensible for TEHVs intended for clinical translation, and the integration of predictive simulations with adaptive conditioning systems represents a critical step toward durable, functional, and scalable living valve replacements.

## 6. Emerging Technologies

New technologies are increasingly expanding TEHV development by building on advances in biomimetic scaffolds, biological cues, and mechanical conditioning. Among the most promising emerging approaches are 3D bioprinting, smart biomaterials, and image-guided tissue engineering, combined with digital twin frameworks, all aiming to improve the precision, and translational potential of engineered valves.

**Figure 9:**
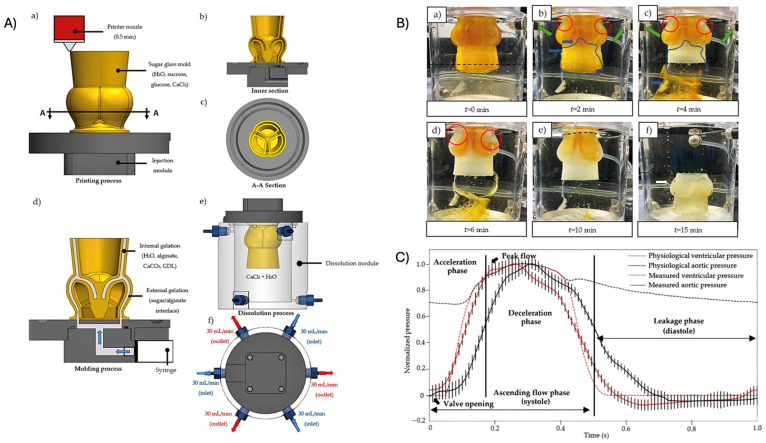
(**A**) Schematic of aortic valve scaffold fabrication. (a) Sugar glass aortic valve mold loaded with CaCl_2_ and printed using a 0.5 mm nozzle and 0.2 mm layer height. (b) Inner section of the mold. (c) A–A cross-section showing the interior at the mid-sinus. (d) Molding of the scaffold by syringe injection along the direction indicated by the arrow of a sodium alginate formulation containing GDL and CaCO_3_ for internal gelation; external gelation occurred at the sugar–alginate interface. (e) Transfer of the injected mold to the dissolution module. (f) Sugar mold dissolution using a 90 mL/min total (inlet/outlet) flow of water supplemented with CaCl_2_. (**B**) Dissolution of the sugar glass mold and final scaffold gelation. (a) t = 0 min, fresh CaCl_2_ solution above the dashed line and dissolved sugar at the bottom (yellowish liquid). (b) t = 2 min, inlet jets of fresh CaCl_2_ (green arrows) created holes in the sugar glass mold (red circles); blue arrows and outlined regions indicate dissolving inner structures falling to the bottom. (c) Mold dissolution at t = 4 min. (d) Mold dissolution at t = 6 min. (e) t = 10 min, scaffold manually cut with a scalpel at the dotted line. (f) t = 15 min, dissolution and gelation completed. (**C**) Average measured pressures (aortic and ventricular) normalized (*n* = 12 cardiac cycles) at 60 BPM. These were obtained using a custom-made cardiac bioreactor on three aortic valve scaffolds and compared to the physiological profiles. The vertical bars represent the standard deviation at each acquisition time (100 Hz) of the average profiles of the three aortic valve scaffolds. Adapted from Rioux et al. [98], *Int. Jour of Mol- Scienc.*, 2022, licensed under CC BY 4.0.

3D bioprinting enables layer-by-layer assembly of spatially controlled cell-laden constructs allowing patient-specific geometries that approximate native leaflet architecture. Customized alginate-based hydrogel valve scaffolds, for example, have demonstrated reproducible leaflet-like geometries suitable for in vitro evaluation [98] (Figure 9). However, current bioinks often lack sufficient mechanical strength and require post-printing conditioning or hybrid reinforcement to achieve functional competence.

In parallel, smart biomaterials introduce stimulus-responsive functionality into scaffold systems. These engineered polymers or composites respond to physiological cues (e.g., pH, enzymatic activity, oxidative stress), enabling adaptive regulation of tissue remodeling. Targeted nanoparticle systems have shown potential in reducing calcification in preclinical models [99]; however, long-term safety, manufacturing reproducibility, and regulatory complexity remain key barriers to clinical translation.

Finally, image-guided tissue engineering integrates advanced imaging modalities with computational modeling to assess valve dynamics and guide scaffold optimization. When coupled with digital twin frameworks, these approaches enable predictive simulations of patient-specific hemodynamics and durability [100]. Although still emerging, such strategies may facilitate predictive, patient-tailored TEHV design.

Following the analysis of these emerging strategies, Table 3 provides a comprehensive synthesis of recent TEHV research milestones and biofabrication advances, highlighting the ongoing paradigm shift toward hybrid, multilayered constructs capable of combining immediate mechanical performance with long-term biological remodeling.

## 7. Challenges Toward Clinical Translation

Despite significant progress in TEHV technologies, considerable challenges remain in their transition to clinical applications. These challenges involve technical, biological, and regulatory issues that must be solved to ensure safe and durable engineered valves.

### 7.1. Scalability and Manufacturing Reproducibility

One of the primary challenges concerns scalability and manufacturing reproducibility. As TEHV designs become increasingly complex maintaining batch-to-batch consistency becomes more difficult. As detailed by Ganizada et al. [103], maintaining consistency across batches while scaling up production presents risks related to mechanical properties, biocompatibility, and overall tissue function.

To overcome these barriers, the field must transition toward automated, closed-loop biofabrication platforms that integrate real-time quality control sensors. Standardizing “off-the-shelf” acellular scaffolds—such as those produced by high-throughput electrospinning or melt electrowriting (mew)—can significantly reduce the variability inherent in patient-specific cell expansion while ensuring structural stability [92,94].

Implementing well-defined protocols and standardized approaches in bioreactor design is crucial for achieving the consistent quality necessary for clinical use.

### 7.2. Vascularization and Integration

Vascularization and integration of TEHVs within host tissues also pose significant challenges. The ability of engineered valves to integrate with surrounding vascular networks is vital for nutrient delivery and tissue viability. Actionable strategies to accelerate this process include the functionalization of scaffolds with pro-angiogenic cues, such as vascular endothelial growth factor (VEGF) or growth differentiation factor 11 (GDF11), which have been shown to enhance endothelial progenitor cell (EPC) recruitment. Furthermore, the use of perfusion-based pre-conditioning within bioreactors can stimulate the formation of primitive vascular-like networks and maintain ECM integrity before implantation [72,73]. Recent studies emphasize that effective vascular networks must be developed within TEHVs to prevent ischemic conditions post-implantation. As demonstrated by Tomiyama et al. [104], using perfusion bioreactors that mimic physiological flows can enhance vascularization while supporting the maintenance of scaffold architecture and ECM integrity.

Without sufficient vascular networks, long-term success of TEHVs is jeopardized as they may fail to receive necessary blood supply in vivo.

### 7.3. Long-Term Durability and Regulatory Pathways

Ensuring the long-term durability of TEHVs in vivo remains another significant concern. Issues such as calcification, immunogenic responses, and mechanical fatigue can lead to the failure of engineered constructs under physiological conditions. A comprehensive understanding of the long-term performance of TEHVs is necessary for advancing these technologies into the clinical arena. To address these failure modes, future roadmaps prioritize hybrid architectures that combine the mechanical robustness of synthetic polymers like polycaprolactone (PCL) with the biological resilience of natural hydrogel coatings, such as chitosan methacryloyl (Chs-MA), which have been shown to suppress calcification and improve hemocompatibility [13]. Studies by Stefano et al. reviewed durability and compatibility issues associated with various scaffolding materials, indicating the need for developing strategies that enhance the resilience and longevity of engineered heart valves in a dynamic biological environment [105].

In addition to technical and biological considerations, regulatory and ethical challenges play a major role in limiting clinical translation. Navigating regulatory and ethical hurdles further significantly complicates the clinical translation of TEHVs. Regulatory bodies require extensive data on safety and efficacy from both preclinical and clinical trials before approving these innovative therapies for patient use. TEHVs often combine advanced biomaterials, living cells, bioactive molecules, and dynamic conditioning systems, resulting in complex products that do not fit neatly within existing regulatory frameworks. The lack of standardized benchmarks for preclinical testing, conditioning protocols, and long-term performance metrics further complicates regulatory approval pathways, increasing development time and cost [106]. To streamline regulatory approval, the development of “in silico clinical trials” using digital twins—virtual representations of patient-specific anatomy—can provide standardized, predictive performance data for large “virtual patient cohorts,” potentially reducing the burden of costly animal testing. Establishing consensus-based benchmarks for dynamic conditioning—specifically aligned with ISO 5840 standards for effective orifice area and regurgitation—is a critical next step for the field to ensure performance metrics are met before human trials [45,95].

From a surgical perspective, scaffold suturability and anchoring remain critical translational requirements. TEHV constructs must provide sufficient suture retention strength to prevent leaflet tearing or delamination during implantation, particularly for hydrogel-rich or rapidly degrading materials. Hybrid reinforcement strategies are therefore often necessary to ensure operative handling and long-term fixation [22].

Although conductive biomaterials are gaining attention in broader cardiovascular tissue engineering, their functional relevance in TEHVs is still emerging and requires further investigation [20].

In summary, although emerging TEHV technologies show strong potential, addressing challenges related to scalability, vascularization, regulatory pathways, and long-term durability will be essential for successful clinical translation. Achieving this goal will require coordinated advances in biomaterials, manufacturing strategies, preclinical evaluation, and regulatory alignment.

## 8. Future Directions

Despite significant advances in tissue-engineered heart valve research—particularly in biomimetic scaffold design, dynamic conditioning systems, and improved preclinical models—the field has yet to achieve the level of functional maturity and long-term reliability necessary for widespread clinical application. Current biomimetic approaches often suffer from an incomplete recapitulation of the native valve’s complex hierarchical anisotropy, particularly the interplay between the three distinct layers (fibrosa, spongiosa, and ventricularis). Furthermore, while scaffolds can provide initial mechanical functionality, precisely controlling long-term remodeling behavior and preventing pathological outcomes—such as calcification or leaflet retraction due to excessive myofibroblast activity—remains a major hurdle. Evidence synthesized in the recent literature highlights that biomechanical stimulation, scaffold architecture, and cell–matrix dynamics are inseparable components of successful valve engineering [107]. Future progress will depend on the convergence of these elements into standardized, reproducible, and patient-tailored manufacturing strategies [108].

A key emerging trend is the transition from static scaffolds toward adaptive, mechano-responsive architectures. Adaptive or mechanoresponsive architectures refer to scaffolds designed to dynamically adjust their mechanical properties in response to applied forces or cellular activity, thereby promoting tissue development that mimics the native valve’s biomechanical environment. Intelligent bioreactors are advanced culture systems equipped with real-time sensors and automated control of mechanical and biochemical cues, allowing for continuous monitoring and dynamic adjustment of tissue conditioning to optimize maturation [109]. Emerging manufacturing approaches, including melt electrowriting and gradient electrospinning, enable precise control over scaffold anisotropy and layered microstructures, which can closely mimic the biomechanics of native leaflets [109]. Next generation scaffolds are expected to integrate regions of tunable stiffness, controlled degradation kinetics, and microarchitectural patterns optimized for directional collagen alignment, thereby enhancing tissue functionality [110]. Coupling these designs with in situ sensors to monitor strain, leaflet bending, and hydration states could further enhance bioreactor conditioning, thereby improving the precision of tissue maturation [109].

The maturation phase of TEHVs is increasingly supported by intelligent bioreactors equipped with closed-loop control, multiparametric sensing, and non-invasive imaging. MRI- and ultrasound-compatible bioreactors have demonstrated the feasibility of monitoring, leaflet motion, ECM deposition, and tissue stiffness without disrupting culture conditions [111,112]. Multi-modal conditioning strategies, incorporating additional physical cues like electrical stimulation, may further refine this maturation process. Future platforms are expected to integrate computational models directly into bioreactor control systems enabling dynamic adjustment of mechanical stimuli based on construct development. This approach may reduce variability between laboratories and facilitate regulatory acceptance [113,114].

At the biological level, consensus on the optimal cell source strategy remains elusive. Sources such as mesenchymal stromal cells, endothelial cells, and induced pluripotent stem cell lineages all show promise; however, challenges as phenotypic drift, overactive remodeling, and inflammatory activation persist [115]. Future research must focus on developing controlled differentiation protocols and epigenetic stabilization techniques to ensure predictable cellular behaviors post-implantation [116]. Furthermore, the integration of organoid-like microtissues, lineage-restricted progenitors, or spatially localized cell populations may enhance ECM deposition and valve durability. The exploration of fully acellular scaffolds is still ongoing, with the hope that host-driven repopulation—if biomechanically guided—will yield more stable, patient-adapted tissues [115].

Another critical direction for the future is the translation toward growth-compatible pediatric TEHVs. Achieving constructs that can accommodate somatic growth without structural failure or pathological remodeling remains a significant challenge. Advances in degradable anisotropic scaffolds, controlled immunomodulation, and mechanobiology-informed conditioning may enable the development of long-term pediatric TEHVs, thereby reducing the need for repeated surgeries. Achieving constructs that can accommodate somatic growth without structural failure—specifically by matching scaffold degradation to the rate of new tissue formation—is essential to reduce the need for repeated surgeries in younger populations [117,118].

On the translational front, computational modeling and in silico trials are expected to become increasingly important. Advanced simulation tools—such as high-fidelity fluid–structure interaction models, growth and remodeling algorithms, and machine-learning-based predictive methods can help to predict how leaflets deform, where stresses are concentrated, and how fatigue may develop in response to patient-specific hemodynamics [119,120]. These digital twins offer the potential to optimize design before the first incision is ever made [113,121].

Finally, key barriers to clinical translation remain, including variability in scaffold manufacturing, limited long-term in vivo data, and the lack of standardized benchmarks for dynamic conditioning. Addressing these issues will require shared conditioning protocols, defined implantation criteria, and comparative studies to enable reliable clinical translation [122,123].

## 9. Conclusions

In conclusion, the development of TEHVs has moved beyond simple material selection toward a sophisticated, integrated engineering paradigm. The main take-home message of this review is that successful clinical translation requires the synchronized control of scaffold anisotropy, immune modulation, and mechanical conditioning rather than incremental material optimization. Biomimetic design serves as the cornerstone of this approach, providing the structural and biochemical cues necessary to guide host–cell behavior toward regeneration rather than fibrosis [124,125].

However, significant limitations still restrict the leap from the bench to the bedside. Current designs frequently fail to fully replicate the adaptive and regenerative properties of native valves, often leading to long-term failure modes like calcification or structural deterioration. Furthermore, key barriers such as manufacturing variability, the lack of standardized benchmarks for dynamic conditioning, and the complexity of navigating regulatory frameworks for “living” medical devices must be addressed [124,125].

Ultimately, the field is entering a convergent phase where advanced biomaterials, adaptive bioreactors, and predictive modeling are being woven into cohesive workflows. Continued progress in standardizing these technologies and ensuring their scalability will be the defining factor in providing patients with living, growing, and durable heart valve replacements.

## Figures and Tables

**Table 1:** Comparative assessment of primary material classes for tissue-engineered heart valve (TEHV) scaffolds, detailing trade-offs between biological fidelity and engineering control. The evaluation encompasses decellularized extracellular matrix (dECM), synthetic polymers, and natural polymers based on their biocompatibility, degradation kinetics, and mechanical resilience under physiological hemodynamic loading.

Material Classes	Advantages	Disadvantages
**Decellularized ECM (dECM)** (e.g., *Porcine/bovine valves*)	Superior structural fidelity. Retains native ECM composition. High initial integrity and native anisotropy.	Risks of residual immunogenicity. Potential for calcification Decellularization process can compromise tensile strength.
**Synthetic Polymers** (e.g., *PGA*, *PCL*, *PLA*, *PGS*)	High tensile strength. Able to withstand physiological hemodynamic loads. Tunable degradation kinetics (rapid to slow).	Limited intrinsic bioactivity. Requires surface modification for cell adhesion.
**Natural Polymers** (e.g., *Collagen*, *Fibrin*, *GelMA*, *HA*)	High biological compatibility. Provides an optimal niche for tissue regeneration. Susceptible to natural remodeling (MMPs).	Relatively low mechanical performance Typically requires reinforcement with synthetic backbones.

**Table 2:** Comparative evaluation of primary and alternative cell sources utilized in heart valve tissue engineering. The table outlines the physiological roles and strategic advantages of each cell type—ranging from native valvular populations (VICs and VECs) to clinically accessible alternatives (HUVECs and dermal fibroblasts)—while highlighting the limitations regarding phenotypic stability, sourcing, and pathological activation risks in the context of long-term valve remodeling.

Cell Type	Role in TEHV	Advantages	Disadvantages and Limitations
**Valvular Interstitial Cells** (VICs)	Primary matrix-producing cells; distributed throughout all layers.	High biological fidelity; superior production of collagen, GAGs, and elastin; essential for natural growth and repair.	High risk of pathological activation; can transition to myofibroblasts leading to fibrosis and calcification.
**Valvular Endothelial Cells** (VECs)	Non-thrombogenic lining; regulates homeostasis via paracrine signaling.	Maintains hemostasis; produces nitric oxide (NO) to keep VICs quiescent; specialized to withstand complex shear stress.	Highly specific phenotype; difficult to source and maintain compared to vascular endothelial cells.
**Human Umbilical Vein Endothelial Cells** (HUVECs)	Surrogate endothelial lining.	Easily isolated and cost-effective; forms continuous monolayers that improve hemocompatibility and prevent early thrombosis.	Does not fully recapitulate the specialized phenotype or morphology of native VECs.
**Dermal Fibroblasts**	Alternative matrix producer for scaffold recellularization.	Abundant ECM production; highly responsive to mechanical stimuli; offers a scalable alternative to native valve-derived cells.	Lacks valve-specific signaling; long-term behavior in the cardiac environment may differ from native VIC populations.

**Table 3:** A comprehensive summary of recent advancements in tissue-engineered heart valve (TEHV) research, detailing the specific type of valve reconstructed, the materials utilized, the fabrication processes employed, the biological components (cells and bioactive compounds) investigated, and the primary in vitro or in vivo highlights reported for each study.

Authors (Year)	Type of Valve Reconstructed	Type of TEHV	Materials Used	Scaffold Architecture	Fabrication Process	Cells Used	Bioactive Compounds Added	Drugs Loaded	Tissue Maturation Strategy	Study Type	Highlights
Bai et al. (2025) [82]	Hybrid TEHV	Hybrid TEHV	Decellularized xenogeneic heart valve, oxidized HA, SF-PBA, curcumin	Glycoprotein-like network	One-pot hybridization	N/A	Curcumin	Yes (Curcumin acts as both)	N/A	In vivo	Enhanced antithrombosis, immunomodulation, and reduced calcification post-implantation.
Chen et al. (2023) [28]	Pulmonary Valve	Atrial/Pulmonary Valve	Polyvinyl alcohol, porcine tissue	Dynamic template	Decellularization, PVA encapsulation	HUVECs	N/A	N/A	Post-treatment cytocompatibility achieved	In vitro	Improved processability and mechanical properties of decellularized valves treated with PVA.
Chen et al. (2023) [56]	Heart valve (NiTi-TexVal)	Synthetic Polymeric Heart Valve (PHV) intended as a proof-of-concept for future TEHV applications	Nitinol (NiTi) wire, Polyethylene glycol terephthalate (PET), and Ultra-high molecular weight polyethylene (UHMWPE)	Fiber-reinforced textile composite with NiTi wires oriented in the radial direction within a plain weave fabric matrix	Integrated micro-tube weaving technology followed by thermoforming/molding and 3D-printed assembly	N/A	N/A	N/A	N/A	In vitro experimental research (pulsating flow, PIV) and numerical simulation (Finite Element Analysis)	Mimicking natural collagen, slashing peak stress by 46.15% and ensuring consistent leaflet motion. PET outperformed UHMWPE during the cardiac cycle.
Jesus De Morales et al. (2024) [33]	Trileaflet aortic valve	3D-bioprinted, cell-laden hybrid scaffold	Gelatin methacrylate (GelMA), Polyethylene glycol diacrylate (PEGDA), and Polycaprolactone (PCL)	Multilayered: PCL base sheet (100 µm) reinforced with cell-laden GelMA/PEGDA hydrogel	Extrusion-based 3D bioprinting (layer-by-layer)	iPSC-derived mesenchymal stem cells (iMSCs) matured into Valvular Interstitial-like (VIC-like) cells	N/A	N/A	Dynamic conditioning (shear and stretch) for 14 days to promote ECM synthesis	In vitro (Dynamic culture/bioreactor)	Hybrid scaffold mimics the native aortic valve’s trilayer structure and mechanics. Dynamic culture significantly reduced ACTA2 (indicator of disease/activation). Promoted healthy ECM markers (Vimentin, Collagen I, Aggrecan) over 14 days.
Fan et al. (2026) [101]	Decellularized aortic valve (DAV)	In situ regenerative/immunomodulatory TEHV	DAV, TPA-PVA hydrogel, mesoporous silica nanoparticles (MSNs)	Natural DAV matrix coated with nanoreservoir-laden hydrogel	Decellularization and hydrogel-nanoreservoir surface modification	THP-1 (macrophages), HUVECs (endothelial cells)	Folic acid, Hyaluronic acid	CY-09 (NLRP3 inhibitor)	In situ M2 macrophage polarization	In vitro and In vivo (rat models)	Intelligent ROS-responsive drug release; Induced M2 macrophage polarization; Accelerated endothelialization and remodeling.
Ma et al. (2024) [38]	Heart Valve Leaflet	Trilayer Biomimetic Scaffold	Atelocollagen (Type I), Hyaluronic Acid (HA), Elastin gel	Trilayer structure mimicking the native leaflet’s structure, composition, and anisotropic bending	Fabrication/Characterization	N/A	N/A	N/A	N/A	Characterization/In vitro (aim)	Scaffold mimics the structure, composition, and anisotropic bending properties of native heart valve leaflets.
Motta et al. (2023) [102]	FibraValves (Nanofiber Heart Valves)	Synthetic nanofiber-based heart valve replacement	PLCL (Poly(L-lactide-co-ε-caprolactone)) and PCL (Polycaprolactone)	Micro- and nanofiber scaffolds with anisotropic alignment mimicking native valve mechanics	Focused Rotary Jet Spinning (FRJS)	Host cells (in vivo)	None	None	In situ remodeling (host-mediated)	In vitro testing and acute in vivo deployment	FibraValves (Nanofiber Heart Valves)
Rioux et al. (2022) [98]	Tricuspid aortic valve	Sodium alginate hydrogel scaffold	Sodium alginate and sugar glass sacrificial molds (sucrose, dextrose, calcium chloride)	Native-like tricuspid geometry featuring sinus of Valsalva and sino-tubular junction	3D-printed sacrificial sugar glass molding and dissolution	None in this study (intended for future autologous cell seeding)	None in this study (intended for future ECM protein addition)	None	None (intended for dynamic cardiac bioreactor stimulation)	In vitro Biofabrication and hydrodynamic validation	This method enables the rapid manufacturing of complex tricuspid scaffolds in 25 min with high geometric reproducibility and functional performance mimicking physiological flow profiles.
Wang et al. (2024) [36]	Heart Valve (Aortic)	Nanofiber-reinforced Sandwich Structure	Polylactic Acid (PLA)/Decellularized Porcine Heart Valve (DPHV)	Sandwich Composite: PLA nanofibers/DPHV/PLA nanofibers	Electrospinning (combined with non-woven processing)	HUVECs	Decellularized ECM (natural components)	N/A	In vitro cell culture and assessment of biocompatibility	In vitro	Sandwich structure improved tensile strength by 108% and tensile strain by 571%; optimized spinning time promoted HUVEC proliferation.
Xu et al. (2024) [14]	Heart Valve	Anisotropic Scaffold	PCL/GelMA/ChsMA Hydrogels	Anisotropic PCL scaffold integrated with bioactive hydrogels	Melt electrowriting (MEW)	Valvular Interstitial Cells (VICs)	ChsMA (for hemocompatibility/endothelialization)	N/A	3D culture to foster ECM remodeling of VICs	In vitro/Mechanical testing	MEW created a biomimetic anisotropic PCL scaffold with tunable mechanical properties; ChsMA improved the hemocompatibility and endothelialization.
Yu et al. (2023) [85]	Heart Valve	Biofunctionalized DTEHV	DTEHV/Mesoporous Silica Nanoparticles (MSNPs)	Decellularized ECM	Biofunctionalization/Drug Loading onto DTEHV	N/A	VEGF (Vascular Endothelial Growth Factor)	RunX2-siRNA	Controlled release of therapeutics to inhibit calcification	Implied In vitro/Pre-clinical	Used MSNPs for controlled, simultaneous release of VEGF and RunX2-siRNA to combat calcification.
Zhang et al. (2025) [47]	Surgical heart valve	Bioinspired trilayer tissue-engineered heart valve (TEHV) scaffold	Poly(e-caprolactone) (PCL)	Trilayer structure with native-like corrugations and orthogonal fiber alignment	Electrospinning using roller and honeycomb-patterned collectors	A7r5 (smooth muscle cells) and HUVECs (endothelial cells)	None reported	None reported	In vitro cell culture for alignment and proliferation	In vitro experimental research and finite element modeling	This biomimetic trilayer scaffold successfully replicated the native “J-curve” stress–strain behavior and mechanical anisotropy of natural leaflets. Hemodynamic testing confirmed the valve meets ISO 5840-2 standards for effective orifice area and regurgitation.

Key to abbreviations: BHV (bioprosthetic heart valves); BP (Bisphosphonate); ChsMA (chitosan methacryloyl); Cu (Copper ions); DAVs (Decellularized Aortic Valves); DHV (decellularized heart valves); DPHV (decellularized porcine heart valves); DPP (decellularized porcine pericardium); DPVs (Decellularized Porcine Valves); DTEHV (Decellularized Tissue-Engineered Heart Valve); ECM (extracellular matrix); FEA (Finite Element Analysis); FRJS (Focused Rotary Jet Spinning); GDF11 (Growth Differentiation Factor 11); HA (hyaluronic acid); HUVECs (human umbilical vein endothelial cells); MEW (melt electrowriting); MSNPs (mesoporous silica nanoparticles); N/A (not applicable/not available); PCL (Poly(ε-caprolactone)); PLA (polylactic acid); PLCL (Poly(L-lactide-co-ε-caprolactone)); PVA (polyvinyl alcohol); REDV (Arginine–Glutamic acid–Aspartic acid–Valine peptide sequence); ROS (Reactive Oxygen Species); RVOT (Right Ventricular Outflow Tract); SF-PBA (Silk Fibroin–Phenylboronic Acid); siRNA (Small interfering RNA); TEHV (tissue-engineered heart valve); VEGF (vascular endothelial growth factor); VICs (valvular interstitial cells).

## Data Availability

No new data were created or analyzed in this study. Data sharing is not applicable to this article.

## Abbreviations

The following abbreviations are used in this manuscript:

The ACTA2	Actin Alpha 2 (Smooth Muscle) gene (α-smooth muscle actin marker)
Arg-1	Arginase-1
AV	Atrioventricular valves
BAV	Bicuspid Aortic Valve
BHV	Bioprosthetic Heart Valve
BPM	Beats Per Minute
BP	Bisphosphonate
CC BY	Creative Commons Attribution license
CD86	Cluster of Differentiation 86
CD206	Cluster of Differentiation 206
ChsMA	Chitosan Methacryloyl (or Chitosan Methacrylate)
CNP	C-type Natriuretic Peptide
COL-I	Collagen Type I
COL-III	Collagen Type III
CS	Chondroitin Sulphate
Cu	Copper ions
CY-09	CY-09 (NLRP3 inhibitor)
DAV	Decellularized Aortic Valve
DAVs	Decellularized Aortic Valves
DAPI	4′,6-diamidino-2-phenylindole
DPP	Decellularized Porcine Pericardium
DPHV	Decellularized Porcine Heart Valve
DPVs	Decellularized Porcine Valves
DTEHV	Decellularized Tissue-Engineered Heart Valve
ECM	Extracellular Matrix
EPC	Endothelial Progenitor Cell(s)
FEA	Finite Element Analysis
FGF-2	Fibroblast Growth Factor 2
FRJS	Focused Rotary Jet Spinning
GAG	Glycosaminoglycans
GDF11	Growth Differentiation Factor 11
GelMA	Gelatin Methacrylate
GenAI	Generative Artificial Intelligence
HA	Hyaluronic Acid
HUVECs	Human Umbilical Vein Endothelial Cells
iMSCs	induced Pluripotent Stem Cell-derived Mesenchymal Stem Cells
iNOS	inducible Nitric Oxide Synthase
iPSC	induced Pluripotent Stem Cell(s)
ISO	International Organization for Standardization
M1	Pro-inflammatory macrophage phenotype (M1-like)
M2	Reparative/pro-regenerative macrophage phenotype (M2-like)
MCP-1	Monocyte Chemoattractant Protein-1
MEW	Melt Electrowriting
MMPs	Matrix Metalloproteinases
MRI	Magnetic Resonance Imaging
MSCs	Mesenchymal Stem Cells
MSNs	Mesoporous Silica Nanoparticles
MSNPs	Mesoporous Silica Nanoparticles
MVP	Mitral Valve Prolapse
N/A	Not Applicable/Not Available
NiTi	Nitinol (Nickel–Titanium alloy)
NLRP3	NLR family pyrin domain containing 3
NO	Nitric Oxide
P4HB	Poly(4-hydroxybutyrate)
PCL	Poly(ε-caprolactone)
PEGDA	Poly(ethylene glycol) diacrylate
PET	Polyethylene terephthalate
PGA	Polyglycolic Acid
PGS	Poly(glycerol sebacate)
PHV	Polymeric Heart Valve
PIV	Particle Image Velocimetry
PLA	Polylactic Acid
PLCL	Poly(L-lactide-co-ε-caprolactone)
PLLA	Poly-L-lactic Acid
PTEN	Phosphatase and Tensin Homolog
PU	Polyurethane
PVA	Polyvinyl Alcohol
REDV	Arginine–Glutamic acid–Aspartic acid–Valine peptide sequence
RGD	Arginine–Glycine–Aspartic acid peptide motif
ROS	Reactive Oxygen Species
RSS	Reynolds Shear Stress
RVOT	Right Ventricular Outflow Tract
SDF-1α	Stromal cell-Derived Factor 1 alpha
SEM	Scanning Electron Microscopy
SF-PBA	Silk Fibroin–Phenylboronic Acid
siRNA	Small interfering RNA
SLs	Semilunar valves
SVs	Semilunar valves
SVD	Structural Valve Deterioration
TE	Tissue Engineering
TEHV	Tissue-Engineered Heart Valve
TEHVs	Tissue-Engineered Heart Valves
TGF-β	Transforming Growth Factor beta
TIMP	Tissue Inhibitor of Metalloproteinase
TIMPs	Tissue Inhibitors of Metalloproteinases
TKE	Turbulent Kinetic Energy
TPA-PVA	TPA–PVA hydrogel
UHMWPE	Ultra-high molecular weight polyethylene
VE-cadherin	Vascular Endothelial cadherin
VECs	Valve Endothelial Cells
VEGF	Vascular Endothelial Growth Factor
VHD	Valvular Heart Disease
VICs	Valvular Interstitial Cells
VSS	Wall Shear Stress
